# Mild and metal-free chemical recycling of polyureas via acylation-activated bond cleavage

**DOI:** 10.1093/nsr/nwaf430

**Published:** 2025-10-11

**Authors:** Enbo Yu, Xingbo Liu, Shuo Hou, Yunqin Cai, Minghui Zhu, Jie Chen, Wang Xiao, Zebing Zeng, Bo Qin

**Affiliations:** State Key Laboratory of Chemo and Biosensing, College of Chemistry and Chemical Engineering, Hunan University, Changsha 410082, China; State Key Laboratory of Chemo and Biosensing, College of Chemistry and Chemical Engineering, Hunan University, Changsha 410082, China; State Key Laboratory of Chemo and Biosensing, College of Chemistry and Chemical Engineering, Hunan University, Changsha 410082, China; State Key Laboratory of Chemo and Biosensing, College of Chemistry and Chemical Engineering, Hunan University, Changsha 410082, China; State Key Laboratory of Chemo and Biosensing, College of Chemistry and Chemical Engineering, Hunan University, Changsha 410082, China; State Key Laboratory of Chemo and Biosensing, College of Chemistry and Chemical Engineering, Hunan University, Changsha 410082, China; State Key Laboratory of Chemo and Biosensing, College of Chemistry and Chemical Engineering, Hunan University, Changsha 410082, China; State Key Laboratory of Chemo and Biosensing, College of Chemistry and Chemical Engineering, Hunan University, Changsha 410082, China; State Key Laboratory of Chemo and Biosensing, College of Chemistry and Chemical Engineering, Hunan University, Changsha 410082, China

**Keywords:** polyurea, chemical depolymerization, closed-loop recycling, metal-free catalysis, urea bond cleavage

## Abstract

Chemical recycling of polymers offers a promising strategy to convert post-consumer waste into reusable monomers and chemicals. However, the inherent stability of covalent bonds in many durable polymers, such as polyureas, renders their chemical deconstruction particularly challenging. Herein, we report a mild, efficient and metal-free method for the depolymerization and recovery of both linear and cross-linked polyureas via an acylation-activated urea bond cleavage strategy. Treatment with low-cost, commercially available acetic anhydride and sulfuric acid under ambient pressure transforms robust urea linkages into labile acetylurea intermediates in high yield, which subsequently undergo efficient alkaline hydrolysis to regenerate the pristine diamine monomers. Notably, the reclaimed monomers can be directly repolymerized into polyurea materials with chemical structures and mechanical performance comparable to those of the virgin counterparts. Techno-economic analysis and life-cycle assessment further underscore the economic feasibility and environmental advantages of this approach over conventional recycling methods. This work provides a new and sustainable strategy for polymer recycling under mild conditions and advances the circular economy of durable polymeric materials.

## Teaser

A mild, metal-free method using commercial chemicals breaks down durable polyurea materials into their original monomers for closed-loop recycling into performance-equivalent products.

## INTRODUCTION

Polymers are indispensable in modern society; however, their persistent accumulation and inadequate end-of-life management have led to severe environmental concerns [1–4]. In response, chemical depolymerization and recycling strategies aimed at recovering monomer raw material for the production of virgin-quality polymers have garnered growing attention [5–17]. The success of such approaches critically depends on the development of efficient depolymerization pathways, particularly for polymers featuring robust main-chain linkages [18–23]. Polyureas, typically synthesized via catalyst-free polyaddition reaction of di- or multifunctional isocyanates and amines, exemplify such durable systems [24]. Owing to their exceptional chemical and thermal stability, polyureas find widespread applications in fibers, coatings and adhesives across the automotive, marine, aerospace and construction industries [25]. As a result, global demand for polyureas continues to rise, with the market size projected to reach 1.4 billion USD by 2030 [26]. Nevertheless, in contrast to ester, urethane or amide linkages commonly found in polymers, urea bonds are among the most stable chemical bonds against further reactions including hydrolysis and aminolysis due to the conjugation stabilization effects from their dual amide-like structure and highly ordered intermolecular hydrogen bonding [27]. Therefore, this intrinsic stability, while essential for performance, presents a formidable challenge for degradation and renders polyureas particularly difficult to recycle through conventional chemical methods.

**Figure 1.**
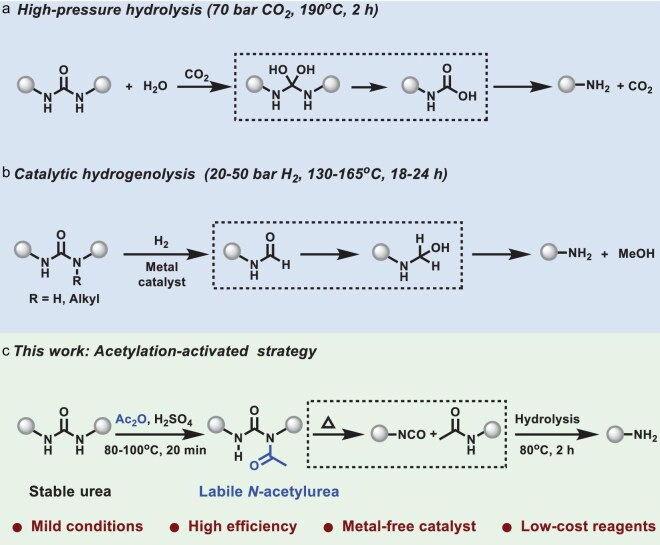
Strategies for the depolymerization of polyureas. (a) High-pressure hydrolysis of polyureas. (b) Metal-catalyzed hydrogenolysis of polyureas. (c) Mild depolymerization and chemical recycling of polyureas via an acylation-activated bond cleavage strategy (this work).

Despite growing interest in polymer recycling, the development of effective depolymerization strategies for polyureas remains limited, particularly when compared to the wide range of approaches available for other condensation polymers—such as pyrolysis, solvolysis and aminolysis [28–38]. Current depolymerization methods for polyureas primarily involve hydrolysis and hydrogenolysis [39,41–43]. For instance, Motokucho and co-workers employed supercritical CO_2_ to hydrolyze polyureas into diamines via hemiacetal and carbamic acid intermediates, under harsh conditions of 190°C and 70 bar (Fig. 1a) [39]. Deng and co-workers demonstrated a catalytic degradation pathway using urea and alcohols in the presence of CuO–ZnO composite catalysts [40]. Subsequently, Iwasaki, Liu and Kumar and their co-workers reported the transition metal-catalyzed hydrogenolysis of polyureas, which generated formamide intermediates that were subsequently hydrogenated, and metal-assisted C–N bond cleavage to yield amines (Fig. 1b) [41–43]. While these methods represent important advances, they generally require elevated temperatures, high pressures or metal-based catalysts, which compromise their practicality and environmental sustainability. Moreover, the cost of such processes often surpasses the value of the recovered materials. Recent alternative strategies have been based on the integration of cleavable or dynamic bonds into polyurea backbones to enable reprocessability or degradability [44–48]. However, such chemistries often require complex monomer design and polymerization regulation, which are incompatible with conventional polymers derived from commercially available monomers [49]. Therefore, there remains an urgent need for mild and efficient depolymerization strategies that enable the chemical recycling of unmodified polyureas under sustainable and metal-free conditions.

In this work, we report a metal-free depolymerization strategy for polyureas via acylation-activated urea bond cleavage under mild conditions, enabling efficient recovery of high-value amine monomers and regeneration of performance-retentive polyurea materials (Fig. 1c). Upon treatment with low-cost acetic anhydride in the presence of sulfuric acid, stable urea linkages are readily converted into labile acylurea intermediates, which undergo subsequent hydrolysis under mild alkaline conditions to reclaim pristine amines. This two-step process—acylation followed by hydrolysis—not only enables the selective cleavage of robust urea bonds but also converts polyureas into their monomeric constituents without the need for pre-installed cleavable units. The method is applicable to both linear and cross-linked conventional polyureas derived from commercially available monomers, offering a general platform for the chemical recycling of durable polyurea materials. Notably, the recovered monomers can be directly repolymerized into new polyurea materials with chemical structures and mechanical performance comparable to those of the virgin counterparts. Furthermore, compared with existing recycling methods, this organocatalytic approach demonstrates improved economic viability and lower environmental impact, as supported by comprehensive techno-economic analysis and life-cycle assessment. This work establishes an efficient and sustainable depolymerization strategy for unmodified polyureas, contributing to the advancement of closed-loop polymer recycling.

## RESULTS AND DISCUSSION

### Screening reaction conditions

**Figure 2.**
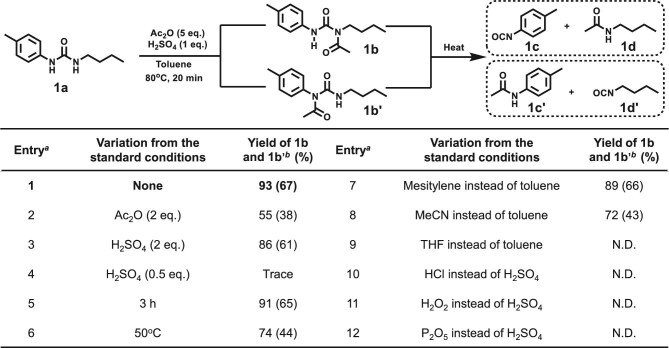
Optimization of acetylation conditions. Note: (a) Standard condition: **1a** (0.1 mmol), Ac_2_O (5.0 equiv.), H_2_SO_4_ (1.0 equiv.), toluene (3 mL), 80^o^C, 20 min, under air. (b) Yields were determined by GC analysis using *n*-tridecane as an internal standard, with the combined yield of **1c′** and **1d** as reference. Isolated yields are shown in parentheses.

To initiate our investigation, 1-butyl-3-(*p*-tolyl)urea (**1a**, Figs S1, S2) was selected as a model substrate for screening reaction conditions (Fig. 2). To our delight, the desired products *N*-butyl-*N*-(*p*-tolylcarbamoyl)acetamide (**1b**) and its regioisomer *N*-(butylcarbamoyl)-*N*-(*p*-tolyl)acetamide (**1b′**) were obtained in excellent yield (93%) when acetic anhydride (Ac_2_O, 5 eq.) and sulfuric acid (H_2_SO_4_, 1 eq.) were used in toluene at 80°C for 20 min (entry 1). Reducing the amount of Ac_2_O led to a substantial decrease in yield (entry 2), indicating that an excess of Ac_2_O is required for efficient conversion. Increasing the H_2_SO_4_ loading slightly reduced the yield, while decreasing the acid loading significantly impaired reactivity, suggesting that an optimal acid concentration is critical for the acylation reaction (entries 3 and 4). Extending the reaction time to 3 h had negligible impact on the yield, indicating that the reaction proceeds rapidly under the optimized conditions (entry 5), while lowering the temperature to 50°C led to a sharp decrease in product formation (entry 6). Solvent screening revealed that mesitylene and MeCN afforded moderate yields, while tetrahydrofuran (THF) suppressed product formation (entries 7–9), highlighting the critical role of solvent in the reaction efficiency. To probe the function of H_2_SO_4_, we performed control experiments using alternative acids or additives. Notably, no product formation was observed in the absence of H_2_SO_4_ or when HCl, H_2_O_2_ or P_2_O_5_ was used instead (entries 10–12). These results indicate that H_2_SO_4_ can effectively activate Ac_2_O toward electrophilic attack, thereby promoting efficient acetylation of the urea substrate.

### Study on acetylation/hydrolysis reaction and mechanism

**Figure 3.**
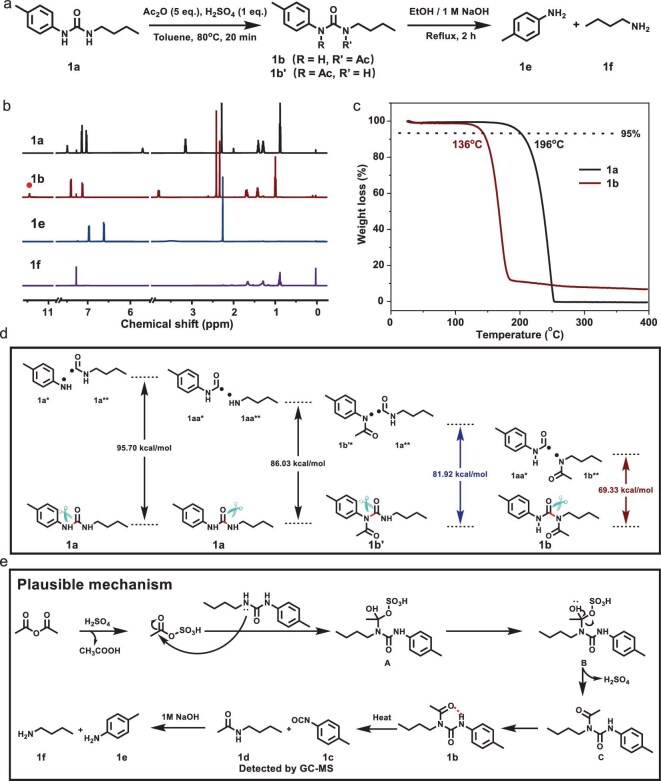
Study on acetylation/hydrolysis reaction and mechanism. (a) Acetylation and subsequent hydrolysis of model compound **1a**. (b) ^1^H NMR spectra of **1a** (top), the acetylation product **1b** (middle) and hydrolysis products **1e** and **1f** (bottom) in CDCl_3_. (c) TGA thermograms of **1a** and **1b** from 25°C to 400°C. (d) Schematic diagram showing BDEs of the C–N bonds (in red) of **1a, 1b** and **1b′**. (e) Proposed mechanism for the acetylation/hydrolysis reaction.

After establishing the optimal acetylation conditions, we next examined the subsequent hydrolysis step to cleave the acetyl group under mild conditions, converting the unstable isocyanate intermediate into amine products. This two-step approach enables both regioisomeric acylated intermediates to converge into a single amine product (Fig. 3a, Fig. S3). The formation of the acylated intermediate (**1b**) and its hydrolysis products (**1e** and **1f**) was confirmed by ^1^H and ^13^C NMR and mass spectrometry (Fig. 3b, Figs S4 and S5). Notably, the appearance of a singlet at δ = 2.304 ppm in the ^1^H NMR spectrum indicates the methyl group of the acetyl moiety, confirming successful acylation. Interestingly, a downfield signal at δ = 11.438 ppm suggests the presence of an intramolecular hydrogen bond in **1b**. This acetylation/hydrolysis strategy was also applicable to diarylureas (e.g. 1,3-di-*p*-tolylurea). Notably, we successfully crystallized the key intermediate *N*-(*p*-tolyl)-*N*′-(*p*-tolylamino)acetamide, whose single-crystal X-ray structure revealed a six-membered intramolecular hydrogen bond between the urea N–H and acetyl C=O, with an O···H distance of 1.865 Å (Tables S12–S18). Subsequent hydrolysis afforded *p*-methylaniline in 87% isolated yield (Figs S9–S11). Thermogravimetric analysis (TGA) further revealed that acetylurea **1b** exhibits significantly reduced thermal stability than the model urea **1a** (Fig. 3c), likely accounting for the absence of its molecular ion peak in the gas chromatography-mass spectrometry (GC-MS) spectrum. To rationalize this thermal lability, we calculated the bond dissociation energies (BDEs) of the key C–N bonds before and after acetylation (Fig. 3d, Fig. S7, Table S1). The BDE of the alkyl-proximal C–N bond in **1b** was calculated to be 69.33 kcal/mol, significantly lower than that of **1a** (86.03 kcal/mol), which can be attributed to electronic and steric effects imparted by the acetyl group. These findings support that the acylation-activated strategy substantially weakens the urea bond, transforming the stable urea bonds into labile acylurea bonds.

Based on the above experimental and computational results, we proposed a plausible reaction mechanism of acylation-activated urea bond cleavage. As shown in Fig. 3e, protonation of Ac_2_O by H_2_SO_4_ generates acetic sulfuric anhydride (detected by LC-MS, Figs S12 and S13), which exhibits markedly enhanced electrophilicity. Additional optimization experiments (Table S2) further support the critical role of H_2_SO_4_ in activating Ac_2_O. This activated species undergoes nucleophilic attack by the urea nitrogen, yielding a key intermediate (**A**). Subsequent collapse of intermediate **A** through elimination of acetic acid and a proton affords the *N*-acylated urea species (**1b**), which features a significantly weakened C–N bond adjacent to the acetyl group. Owing to the electron-withdrawing and steric effects imparted by the acetyl group through electrostatic surface potential calculations (Fig. S8), **1b** is thermally labile and undergoes easy cleavage upon mild heating to generate an isocyanate intermediate (**1c**) and a secondary amide (**1d**), both of which were confirmed by GC-MS analysis (Fig. S6). To verify whether this step proceeds via a homolytic pathway, electron paramagnetic resonance (EPR) spectroscopy revealed no detectable radical intermediates during the thermal cracking process of **1b**, and the reaction rate was unaffected by the radical scavenger 2,2,6,6-tetramethylpiperidin-1-yl)oxyl (TEMPO) (Fig. S14), supporting a heterolytic elimination mechanism. Subsequent hydrolysis of these intermediates under basic conditions produces the corresponding amines (**1e** and **1f**), thereby completing the urea bond cleavage under mild conditions. This acylation-activated bond scission pathway enables efficient urea cleavage under mild, metal-free conditions, offering a promising strategy for the depolymerization and recycling of polyurea materials.

### Depolymerization and recycling of polyureas

**Figure 4.**
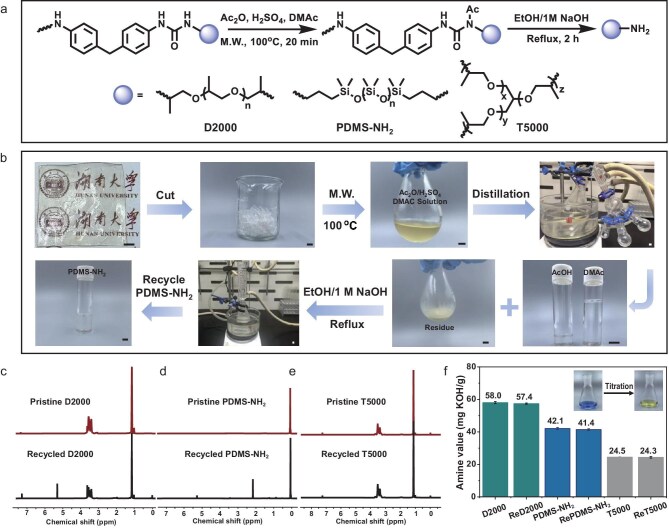
Depolymerization and monomer recovery of polyureas. (a) Schematic illustration of the depolymerization pathways for the three polyureas, including LPU-D2000, LPU-PDMS and CPU-T5000. (b) Photographs showing the depolymerization of LPU-PDMS and recovery of the PDMS-NH_2_ monomer (scale bar: 1 cm). ^1^H NMR spectra of pristine and recycled D2000 (c), PDMS-NH_2_ (d) and T5000 (e). (f) Amine titration of the pristine and recycled D2000, PDMS-NH_2_ and T5000.

**Figure 5.**
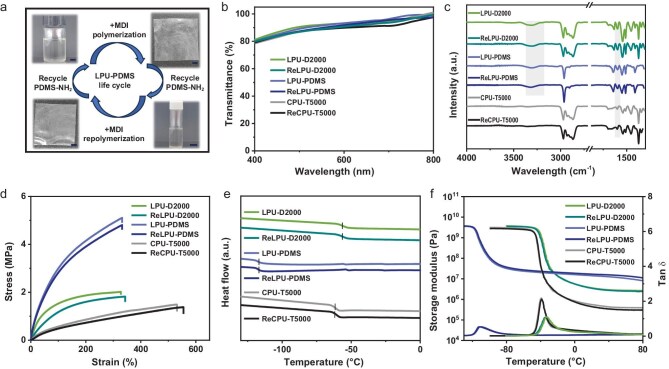
Structural and performance comparison of original and regenerated polyureas. (a) Schematic diagram of the depolymerization–repolymerization cycle of LPU-PDMS. (b) UV-vis transmission spectra, (c) FT-IR spectra, (d) stress–strain curves, (e) DSC curves and (f) DMA curves of the original and regenerated LPU-D2000, LPU-PDMS and CPU-T5000.

Having validated the bond cleavage mechanism using small-molecule model compounds, we next extended the *N*-acetyl substitution strategy to achieve the depolymerization and chemical recycling of polyurea materials. As illustrated in Fig. 4a and Figs S15–S18, three representative linear and cross-linked polyureas were synthesized by reacting 4,4′-methylenebis(phenyl isocyanate) (MDI) and various polyamines, including polyetheramine D2000 (*M_n_* = 2000), polydimethylsiloxane diamine (PDMS-NH_2_) (*M_n_* = 2500) and polyetheramine T5000 (*M_n_* = 5000). Fourier-transform infrared (FT-IR) spectroscopy confirmed the complete conversion of isocyanate groups (disappearance of the −NCO band at 2250 cm^−1^), as well as the formation of polyurea linkages, as evidenced by the characteristic C=O stretching at 1642 cm^−1^ and N–H stretching near 3330 cm^−1^ (Fig. 5c). The polyureas were then *N*-acetylated and activated in *N,N*-dimethylacetamide (DMAc) containing Ac_2_O and H_2_SO_4_, under microwave heating at 100°C for 20 min [50,51]. This activation step was followed by depolymerization in 1 M NaOH ethanol solution at 80°C for 2 h, yielding the original high-value diamine monomers with high purity. Importantly, this depolymerization process is readily scalable. As a demonstration, 4.5 g of lignin-based polyurethane (LPU)-PDMS was dissolved in 10 mL of DMAc, followed by the addition of 773 μL of Ac_2_O and 92 μL of H_2_SO_4_. After microwave-assisted reaction at 100°C, a homogeneous and transparent *N*-acetylated LPU-PDMS solution was obtained. As shown in Figs S19–S22, DMAc and acetic acid were efficiently recovered by distillation (>90% isolated yield, >99% purity). Subsequent hydrolysis of the *N*-acetylated intermediate in ethanol/1 M NaOH aqueous solution afforded PDMS-NH_2_ diamine (60% isolated yield) and 4,4′-diaminodiphenylmethane (MDA) (35% isolated yield). Remarkably, even the cross-linked polyurea (CPU-T5000) was successfully depolymerized by using the same protocol, highlighting the broad applicability of this method. All of these polyureas could be recycled and the recovered diamine monomers were characterized by ^1^H NMR, showing peaks indistinguishable from those of the pristine monomers (Figs 4c–e). Additionally, amine value titration confirmed >98% retention of amine functionality (Figs S23–S25), indicating that the recycling process did not compromise the integrity of the terminal groups. The results confirm that chemical depolymerization of polyureas can be successfully achieved though the acylation-activated urea bond cleavage strategy.

Owing to the high purity and preserved functionality, these recycled diamines and triamines were successfully repolymerized with MDI to afford new-generation polyureas—designated ReLPU-D2000, ReLPU-PDMS and ReCPU-T5000, respectively (Fig. 5a). The regenerated polyurea materials displayed the average transmittance of exceeding 90% across the visible range (Fig. 5b), suggesting minimal structural alteration during the chemical recycling process. As shown in Fig. 5c, FT-IR spectra of the regenerated polymers were almost identical to those of the original polyureas, retaining the characteristic urea C=O and N–H absorptions. Furthermore, the mechanical and thermal properties of the original and regenerated polyureas were systematically compared using tensile testing, TGA, differential scanning calorimetry (DSC), and dynamic mechanical analysis (DMA). TGA profiles for all three polymers showed overlapped degradation curves with almost identical 5% weight loss temperatures (Fig. S30). Mechanical testing revealed consistent Young’s modulus, tensile strength and elongation at break before and after recycling (Fig. 5d, Figs S26–S27, Tables S3–S5), demonstrating full recovery of mechanical properties. In addition, as shown in Figs 5e and f, Table S5 and Figs S28, S29, gel permeation chromatography, DSC and DMA curves showed consistent molecular weights, glass transition temperatures and cross-linking densities of the regenerated polyureas, confirming the preservation of structures and viscoelastic properties. Notably, the regenerated CPU-T5000 exhibited comparable elasticity to its pristine counterpart under cyclic loading–unloading tests, both at fixed and increasing strain levels (Fig. S31). The solvent resistance of CPU-T5000 before and after recycling was evaluated through swelling experiments (Fig. S32a). All the samples showed nearly identical mass swelling ratio and residual mass fraction after 24 h without significant dissolution (Fig. S32b), indicating the integrity of the repolymerized cross-linked network. Moreover, hydrolysis tests showed that the recovered CPU-T5000 maintained its mechanical properties without significant degradation after soaking in acidic and alkaline aqueous solutions (Fig. S32c). Finally, we extended this depolymerization strategy to commercial polyureas. Although the purity and yield of the recovered amines decreased, successful monomer recovery highlights the broader applicability and potential universality of this method (Fig. S33). Collectively, these results confirm that the organocatalytic acetylation-activated strategy enables the mild and efficient chemical recycling of both linear and cross-linked polyureas and yields reusable diamine monomers, thus closing the loop for polyureas circularity.

### Life-cycle and techno-economic assessments

**Figure 6.**
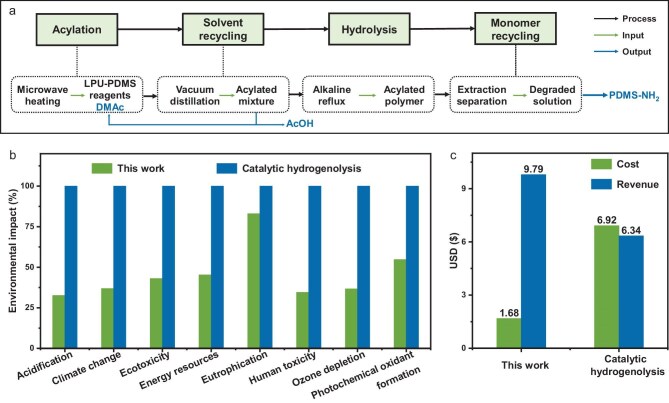
Life-cycle and techno-economic assessments. (a) System boundary for the LPU-PDMS depolymerization process. Comparative results from life-cycle assessment (b) and techno-economic assessment (c).

To further assess the environmental sustainability and economic feasibility of the acylation-activated strategy, we conducted a comprehensive life-cycle assessment (LCA) and techno-economic analysis (TEA) [28,52]. Linear polyurea based on PDMS soft segment (LPU-PDMS) was selected as the representative case owing to the high value of its recovered diamine monomer (PDMS-NH_2_). A simplified process flow diagram is presented in Fig. 6a, showing the process that utilizes efficient microwave-assisted depolymerization method and low-cost, commercially available reaction reagents, including DMAc, Ac_2_O and H_2_SO_4_. Notably, DMAc can be recovered with high purity equivalent to that of fresh solvent, and Ac_2_O can be converted to acetic acid with approximately 90% yield via simple distillation. Most importantly, the recovered diamine monomer can be directly repolymerized into new-generation polyurea materials with structures and properties comparable to the original polymer.

For LCA, conventional metal-catalyzed hydrogenolysis was employed as the benchmark depolymerization process of polyureas. Assuming a single regeneration cycle of LPU-PDMS, the normalized environmental impacts are summarized in Fig. 6b, Tables S6–S11 and Figs S32–S34. Our depolymerization process demonstrates significantly reduced impacts across multiple categories, including acidification (32.6%), climate change (36.9%), ecotoxicity (43.0%), energy resource consumption (45.3%), eutrophication potential (83.0%), human toxicity (34.5%), ozone depletion (36.6%) and photochemical oxidant formation (54.7%). These improvements clearly highlight the reduced environmental footprint of the acylation-activated depolymerization strategy. In addition, from an economic perspective (Fig. 6c), the total cost of the proposed recycling process—including raw materials, energy, labor and capital investment—is estimated at approximately USD 1.68/kg, representing only 24.3% of the cost of conventional hydrogenolysis. Meanwhile, the estimated revenue from the acylation-activated depolymerization process reaches USD 9.79/kg, which corresponds to 149.8% of hydrogenolysis. In summary, this *N*-acylation-enabled depolymerization strategy not only allows for the mild and efficient breakdown of conventional polyureas but also enables closed-loop recycling of high-value monomers into virgin-like materials. Importantly, both LCA and TEA results confirm that this method is economically competitive and environmentally advantageous, offering a compelling route toward sustainable polymer life-cycle management.

## CONCLUSION

In conclusion, we have developed a mild, efficient, and metal-free strategy for the chemical depolymerization of polyureas via acylation-activated bond cleavage. By transforming robust urea linkages into labile *N*-acylated intermediates, this approach enables the recovery of diamine monomers with high purity under mild conditions. Importantly, these recycled monomers can be directly repolymerized to produce new-generation polyureas with chemical structures and mechanical properties comparable to those of the original polyurea materials. TEA and LCA further demonstrate the economic viability and environmental advantages of this approach over other conventional recycling methods. Overall, this work not only offers a practical and sustainable solution for polyureas recycling and circular use, but also lays a chemical foundation for designing dynamic and recyclable urea-based polymer materials.

## METHODS

### Preparation of the linear and cross-linked polyureas

The synthesis of linear polyureas (LPU-D2000, LPU-PDMS, ReLPU-D2000 and ReLPU-PDMS) and cross-linked polyureas (CPU-T5000 and ReCPU-T5000) followed similar procedures, with variations in the choice of polyamine macromonomer. Taking the synthesis of LPU-D2000 as a representative example, polyetheramine D2000 (2.0 g, 1.0 mmol) was dissolved in 20 mL of anhydrous toluene in a 100-mL three-necked round-bottom flask under a nitrogen atmosphere. MDI (0.25 g, 1.0 mmol), separately dissolved in 10 mL of anhydrous toluene, was added dropwise to the polyamine solution at 0°C (approximately one drop per second) under constant stirring. Upon completion of the addition, the reaction mixture was stirred at room temperature for 2 h. For reactions involving PDMS-NH_2_ and T5000, gelation occurred within seconds upon mixing with MDI. The resulting mixture was immediately cast onto a polytetrafluoroethylene (PTFE) dish and dried in a vacuum oven at 80°C for 6–12 h to yield LPU-D2000 films. Prior to characterization, the films were hot-pressed under the following conditions: plate distance = 0.2 mm, temperature = 180°C and pressing time = 5 min.

### Chemical depolymerization and recycling of polyureas

Used LPU-PDMS films (4.5 g) were cut into small pieces and immersed in 10.0 mL of DMAc. Ac_2_O (835.0 mg, 5 equiv.) and sulfuric acid (168.8 mg, 1 equiv.) were added, and the mixture was subjected to microwave heating at 100°C for 20 min. After cooling to room temperature, DMAc and the byproduct acetic acid were recovered via vacuum distillation. The residue was treated with 20.0 mL of 1 M NaOH aqueous solution and 20.0 mL of ethanol, followed by refluxing at 80°C for 2 h. Upon cooling, volatile components were removed under reduced pressure. The resulting mixture was diluted with dichloromethane (15.0 mL) and washed with saturated aqueous NaCl solution. The aqueous phase was extracted with dichloromethane (3×), and the combined organic layers were dried over anhydrous MgSO_4_. After solvent removal under reduced pressure, the crude product was purified by column chromatography on alkaline silica gel (eluent: acetone/ethyl acetate = 1/5) to afford PDMS-NH_2_ as a transparent liquid. The depolymerization and separation procedures for LPU-D2000 and CPU-T5000 followed the same protocol as that of LPU-PDMS.

## Supplementary Material

nwaf430_Supplemental_File
