# Supplementary material for: Mild and metal-free chemical recycling of polyureas via acylation-activated bond cleavage
Source: Natl Sci Rev. 2025 Oct 11;12(12):nwaf430. doi: 10.1093/nsr/nwaf430 (PMC12673572; doi:10.1093/nsr/nwaf430)
Supplement: nwaf430_Supplemental_File [file nwaf430_supplemental_file.pdf]

Supplementary Information

Mild and Metal-Free Chemical Recycling of Polyureas via Acylation-Activated Bond Cleavage

*Enbo Yu[1,2], Xingbo Liu[1,2], Shuo Hou[1], Yunqin Cai[1], Minghui Zhu[1], Jie Chen[1], Wang Xiao[1], Zebing Zeng[1], and Bo Qin[1]**

[1] State Key Laboratory of Chemo and Biosensing, Hunan University, Changsha, 410082, China

[2] These authors contributed equally to this work.

* E-mail: qinbo@hnu.edu.cn (Bo Qin)

Table of Contents

1. Materials and Instrumentation

2. Synthesis and Characterization of Model Compounds

3. Control Experiments

4. Synthesis and Characterization of Polyureas

5. General Experimental Procedure of Depolymerization

6. Mechanical Properties of Pristine and Recycled Polyureas

7. Calculation Data for Life Cycle and Techno-Economic Assessments

8. Crystal Data of 1bb

9. Supplementary References

1. Materials and Instrumentation

1.1 Materials

Poly(propylene glycol) bis(2-aminopropyl ether) (D2000), bis(3-aminopropyl)-terminated poly(dimethylsiloxane) (PDMS2500), poly(propylene glycol) tris(2-aminopropyl ether) (T5000) diphenylmethane diisocyanate (MDI) were obtained from Aladdin Scientific, China. Anhydrous *N*,*N*-Dimethylacetamide (DMAc) was obtained from J&K Scientific, China. All other solvents were supplied by Sinopharm Chemical Reagent Co., Ltd. Commercial polyurea product was supplied by Qingdao Jundun New Materials Co., Ltd. All chemical compounds were used without further purification.

1.2 Instrumentation

^{1}H NMR and ^{13}C spectra were recorded on a Bruker AV-III-400 or a Bruker-600 spectrometer instrument using $CDCl_3$ as solvent and TMS as an internal standard. Mass spectra were measured on Agilent 5977 GC-MS instrument (EI). Liquid chromatograph mass spectrometer (LC-MS) was performed using an Agilent 1260 high-performance liquid chromatography (HPLC) system. High-resolution mass spectra (HRMS) were performed on FTMS ICR MS BRUKER 7T. Fourier transform infrared (FT-IR) spectra were recorded using a UATR Two FT-IR spectrometer equipped with an ATR accessory, within the wavenumber range of 500 to 4000 cm^{-1} at room temperature. The single crystals were grown via slow diffusion of hexane into the chloroform solutions of **1bb** and analyzed with a Bruker D8 Venture X-Ray single crystal diffractometer. Using Olex2, the structures were solved with the SHELXS structure solution program using Direct Methods and refined with the SHELXL refinement package.

Differential scanning calorimetry (DSC) measurements were conducted using a TA instrument Discovery 250 system under a nitrogen flow rate of 50 mL/min. Samples (~10 mg) were heated within a specified temperature range at a rate of 10 ℃/min. To eliminate thermal history effects, the glass transition temperatures were determined from the second heating cycle. Dynamic mechanical analysis (DMA) measurements were conducted using a TA instrument Discovery 850 in tensile mode to evaluate the dynamic mechanical properties of the samples. Rectangular samples with dimensions of 6 mm (length) × 4 mm (width) × 0.2 mm (thickness) were subjected to oscillatory strain while being heated within a specified temperature range at a heating rate of 3 ℃/min and a frequency of 1 Hz. Thermogravimetric analysis (TGA) was performed using a TA Q600-SDT (TA Instruments). The polymer samples were heated under a nitrogen atmosphere from ambient temperature to 600 ℃ or 1000 ℃ at a heating rate of 20 ℃/min.

The stress-strain curves of the polymers were measured using a SANS EUT6502 electric universal testing machine at room temperature. Polymer samples with dimensions of 30 mm (length) × 4 mm (width) × 0.2 mm (thickness) were tested with a gauge length of 10 mm at a cross-head speed of 5 mm/min. Young's modulus values were determined by fitting the slope of the stress-strain curve in the initial linear region. Data were averaged from at least three specimens to ensure accuracy.

2. Synthesis and Characterization of Model Compounds

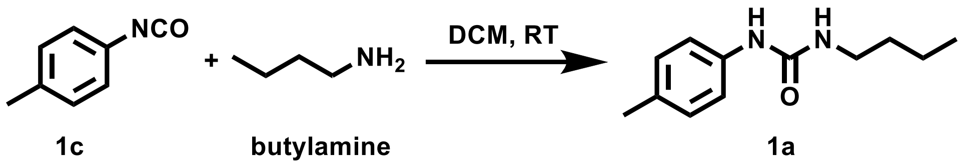

Supplementary Figure 1. Synthetic route of the model urea **1a**.

Exactly 1.26 mL (1.331 g, 10.0 mmol) of phenyl isocyanate **1c** was added dropwise to a solution of butylamine (731.4 mg, 10 mmol) in 20 mL of dry CH_2Cl_2 at room temperature. The resulting solution was stirred for 10 minutes at room temperature and then precipitated into 25 mL of *n*-hexane The product 1a was isolated as a white powder via filtration and washed with 20 mL of *n*-hexane before being dried under vacuum at 50 ℃, yielding a white solid with 97% yield. ^{1}H NMR (600 MHz, $CDCl_3$) δ 7.48 (s, 1H), 7.13 (d, *J* = 8.3 Hz, 2H), 7.02 (d, *J* = 8.1 Hz, 2H), 5.67 (s, 1H), 3.19-3.07 (m, 2H), 2.26 (s, 3H), 1.36-1.41 (m, 2H), 1.23-1.29 (m, 2H), 0.85 (t, *J* = 7.3 Hz, 3H).

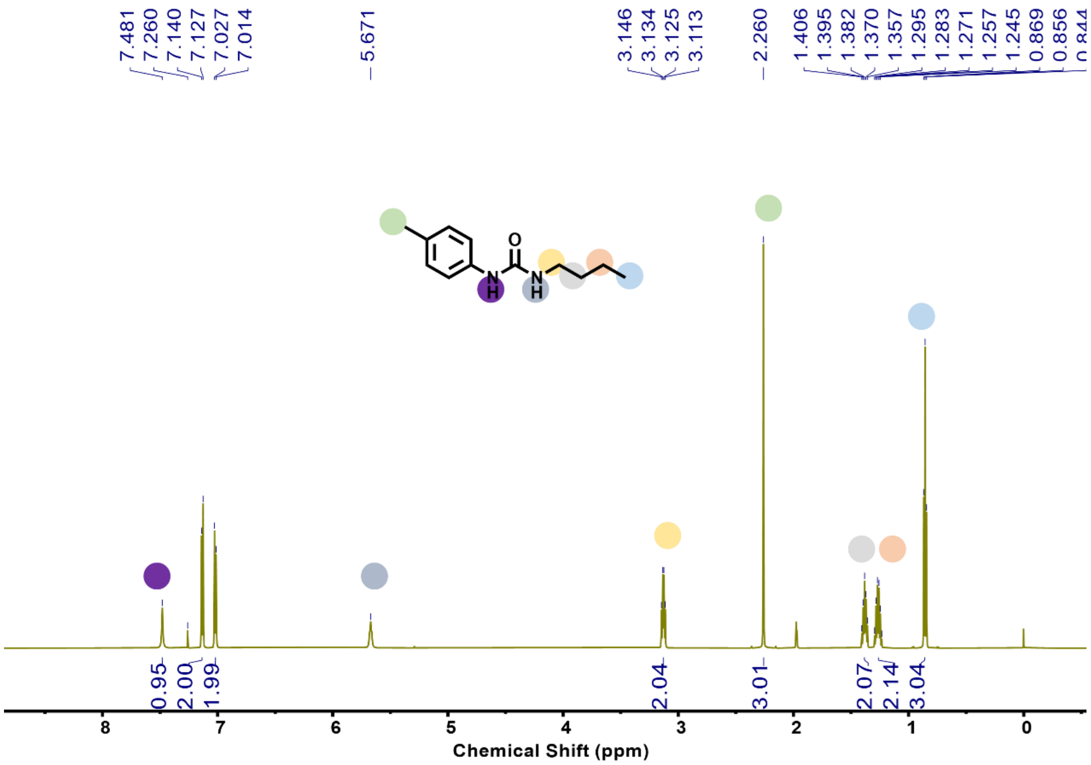

Supplementary Figure 2. ^{1}H NMR Spectrum (600 MHz) of 1a in $CDCl_3$.

3. Control Experiments

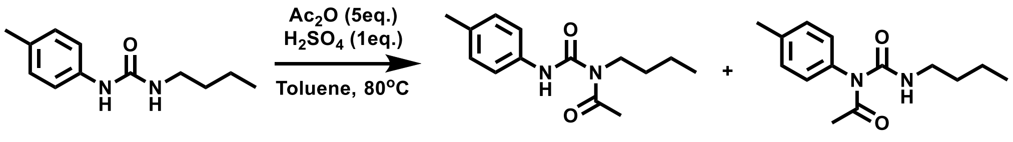

Supplementary Figure 3. Control experiments to capture intermediate products.

1-butyl-3-(*p*-tolyl)urea (206 mg, 1mmol), Ac_2O (472.0 μL, 0.5 mmol), H_2SO_4 (53.0 μL, 1 mmol) and toluene (10 mL) were added to a reaction vessel (25 mL). The reaction was stirred in the oil bath at 80 ℃ under air for 20 min. After cooling to room temperature, the reaction mixture was diluted with ethyl acetate (15.0 mL) and washed by saturated sodium chloride solution. The organic layer was separated and the aqueous layer was extracted with ethyl acetate for three times [1]. The combined organic layer was dried over $MgSO_4$ and the volatiles were removed under reduced pressure. After solvent removal under reduced pressure, the crude product was purified by column chromatography on alkaline silica gel (eluent: EA/PE = 1:5) to afford 1b as a transparent liquid. The consequence was detected by HRMS and GC-MS. HRMS m/z $[M+H]^+$ calcd for $C_{14}H_{21}N_2O_2^+$ 249.1598,found 249.1590.

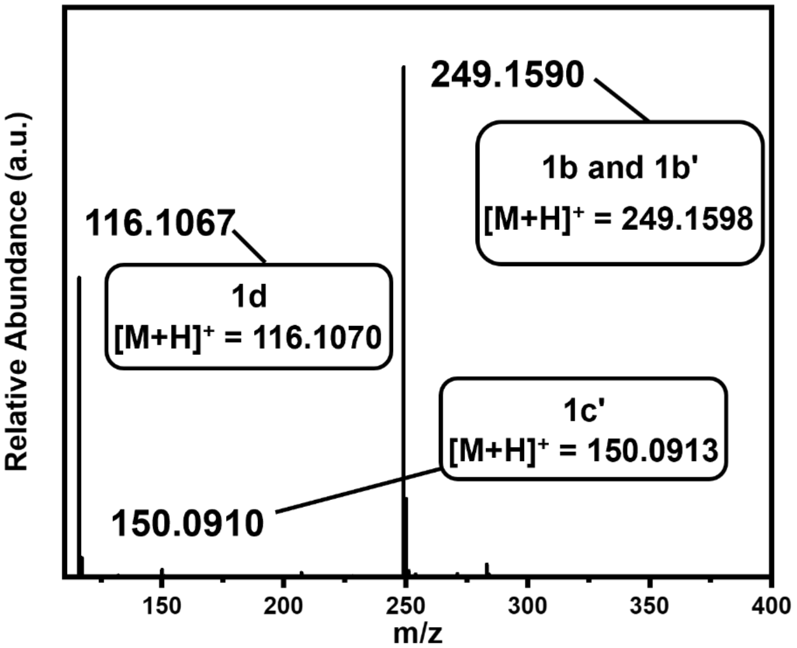

Supplementary Figure 4. HRMS spectrum of intermediate products 1b,1d and 1c'.

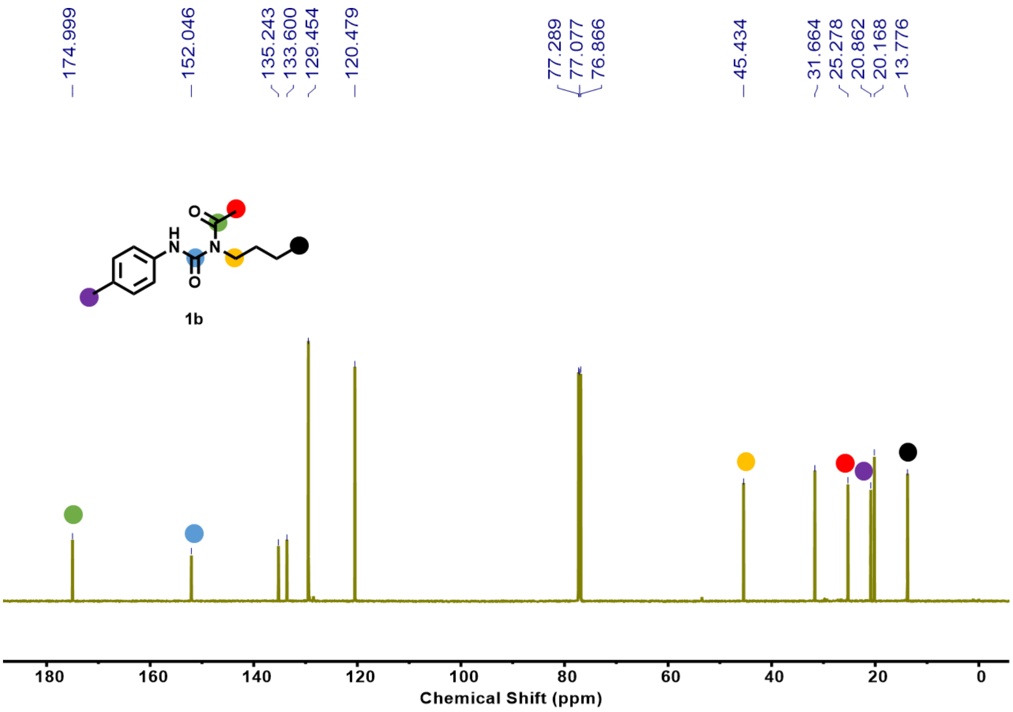

Supplementary Figure 5. ^{13}C NMR (151 MHz) of intermediate product 1b.

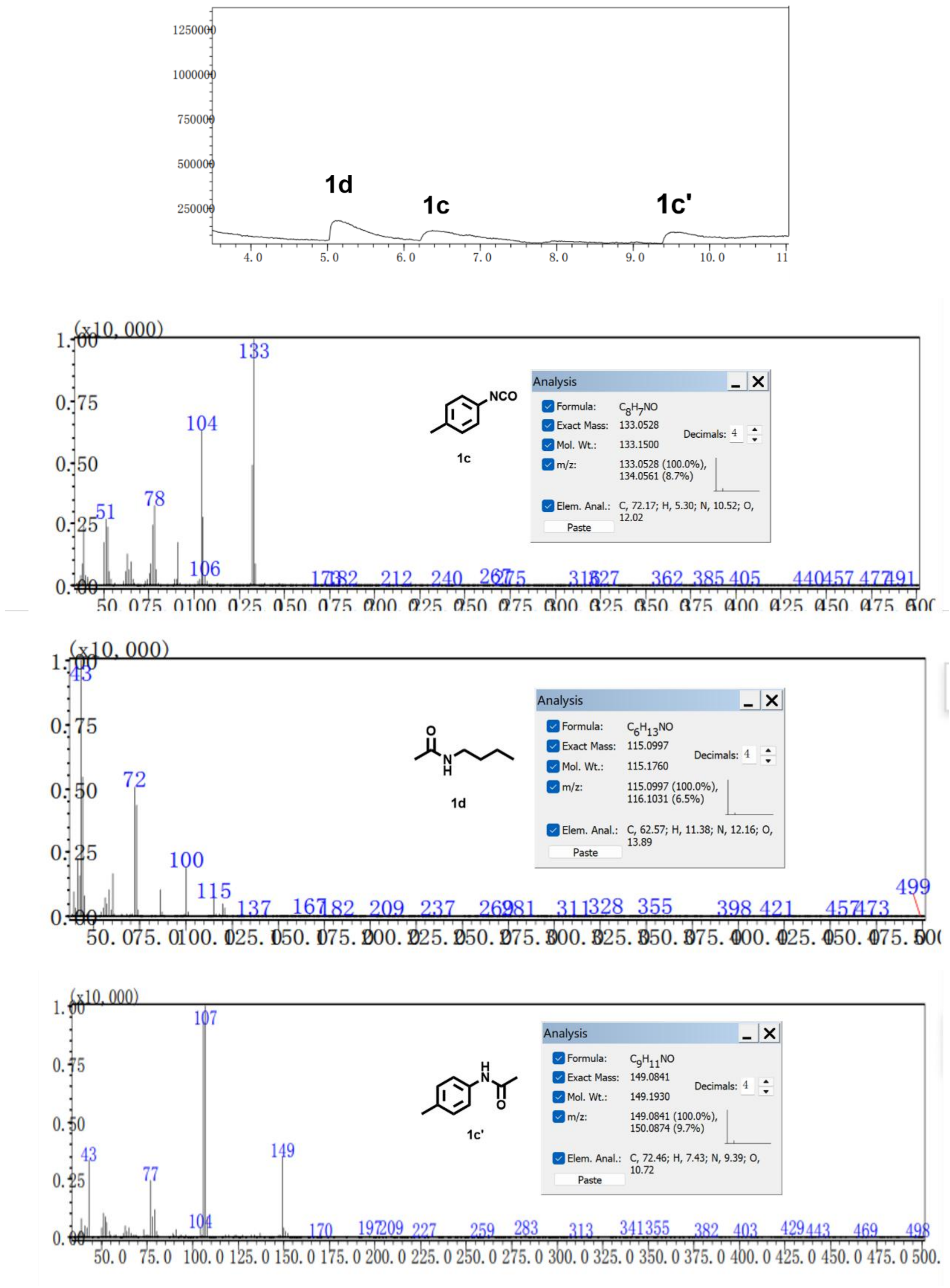

Supplementary Figure 6. GC-MS spectra of the intermediate products.

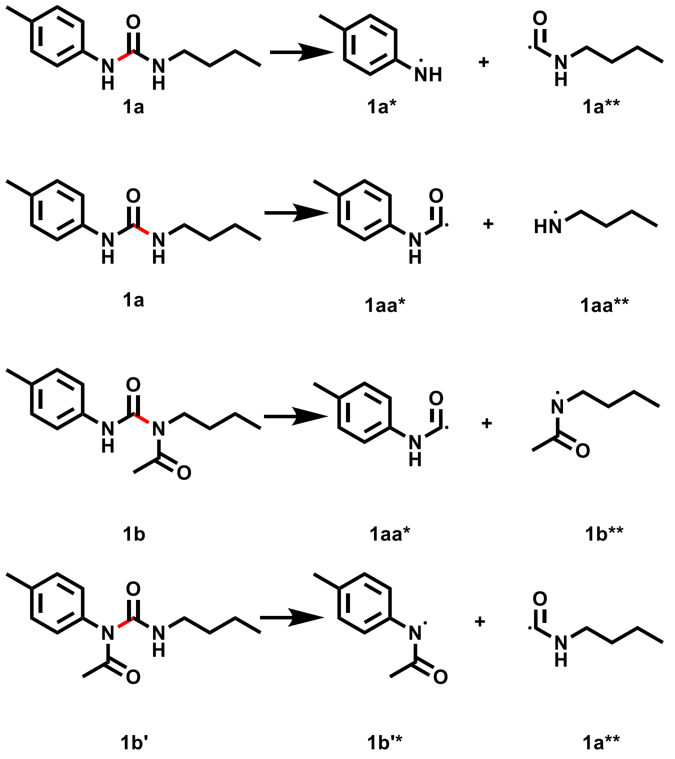

Supplementary Figure 7. Schematic diagram of C-N bond cleavage in small molecule urea.

Supplementary Table 1. Calculate the energy required to break the C-N bond (red) at the B3LYP/def2tzvp D3 level.

**Name**	**Molecular formula**	$\triangle_f H_{298}$	**Unit**
**1a**	$C_{12}H_{18}N_2O$	-652.862759	Hatree
**1a***	$C_7H_8N\cdot$	-326.538983	Hatree
**1a****	$C_5H_{10}NO\cdot$	-326.476331	Hatree
**1aa***	$C_8H_8NO\cdot$	-439.637468	Hatree
**1aa****	$C_4H_{10}N\cdot$	-213.-88197	Hatree
**1b**	$C_{14}H_{20}N_2O_2$	-805.537745	Hatree
**1aa***	$C_8H_8NO\cdot$	-439.637468	Hatree
**1b****	$C_6H_{12}NO\cdot$	-365.769738	Hatree
**1b'**	$C_{14}H_{20}N_2O_2$	-805.533346	Hatree
**1b'***	$C_9H_{10}NO\cdot$	-478.946526	Hatree
**1a****	$C_5H_5NO\cdot$	-326.476331	Hatree

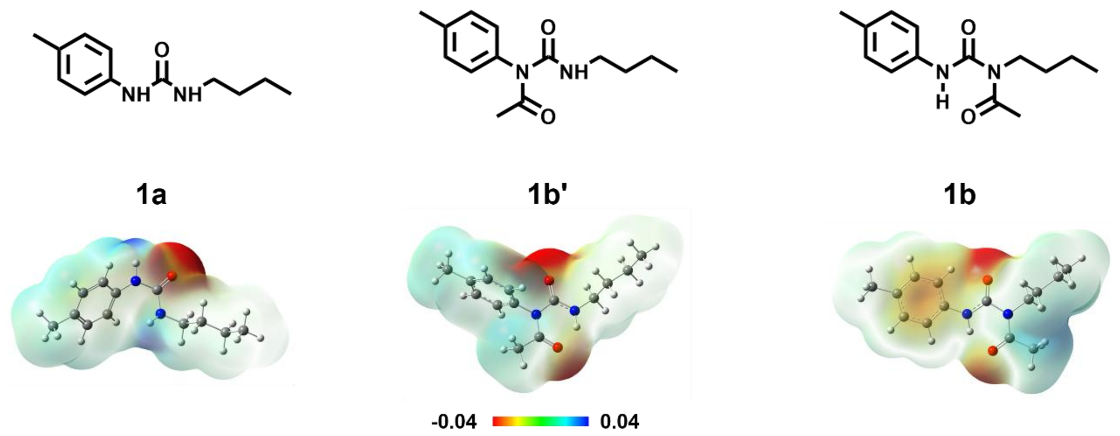

Supplementary Figure 8. The calculated ESP diagrams of 1a, 1b' and 1b.

Supplementary Table 2. Optimization of acetylation conditions. [a] Standard condition: 1a (0.1 mmol), Ac_2O (5.0 equiv.), H_2SO_4 (1.0 equiv.), toluene (3 mL), 80°C, 20 min, under air. [b] Isolated yields.

**Entry**[a]	**Variation from the standard conditions**	**Yield of 1b and 1b'**[b] **(%)**
**1**	none	93(67)
**2**	HCl (1eq.) + Na_2SO_4 (0.5eq.) instead of H_2SO_4	48%
**3**	HCl (1eq.) + $NaHSO_4$ (0.5eq.) instead of H_2SO_4	55%
**4**	H_3PO_4 instead of H_2SO_4	N.D.
**5**	TfOH instead of H_2SO_4	N.D.
**6**	*p*-TsOH instead of H_2SO_4	N.D.

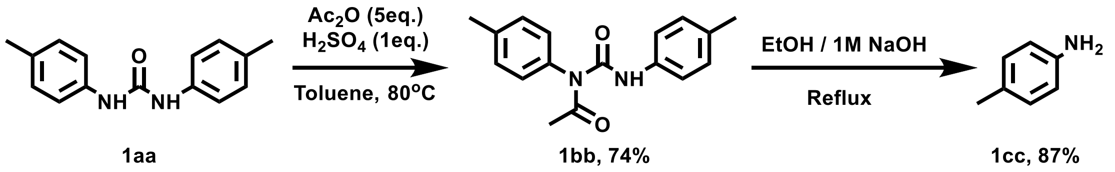

Supplementary Figure 9. Control experiments to explore the applicability of diarylurea.

1,3-di-p-tolylurea (240 mg, 1mmol), Ac_2O (472.0 μL, 0.5 mmol), H_2SO_4 (53.0 μL, 1 mmol) and toluene (10 mL) were added to a reaction vessel (25 mL). The reaction was stirred in the oil bath at 80 ℃ under air for 20 min. After cooling to room temperature, the reaction mixture was diluted with ethyl acetate (15.0 mL) and washed by saturated sodium chloride solution. The organic layer was separated and the aqueous layer was extracted with ethyl acetate for three times. The combined organic layer was dried over $MgSO_4$ and the volatiles

were removed under reduced pressure. After solvent removal under reduced pressure, the crude product was purified by column chromatography on alkaline silica gel (eluent: EA/PE = 1:5) to afford 1bb as a white solid with 74% yield.

Subsequently, 1bb was added to a mixed solvent of 20 mL 1 M sodium hydroxide aqueous solution and 20.0 mL ethanol, and the mixture was refluxed for 2 h. After cooling, the volatile components were removed under reduced pressure. The resulting mixture was diluted with dichloromethane (15.0 mL) and washed with saturated NaCl aqueous solution. The aqueous layer was extracted with dichloromethane (3×), and the combined organic layers were dried over anhydrous magnesium sulfate. After removal of the solvent under reduced pressure, the crude product was purified by silica gel column chromatography (eluent: EA/PE = 1:5) to afford a white solid of *p*-toluidine with 87% yield.

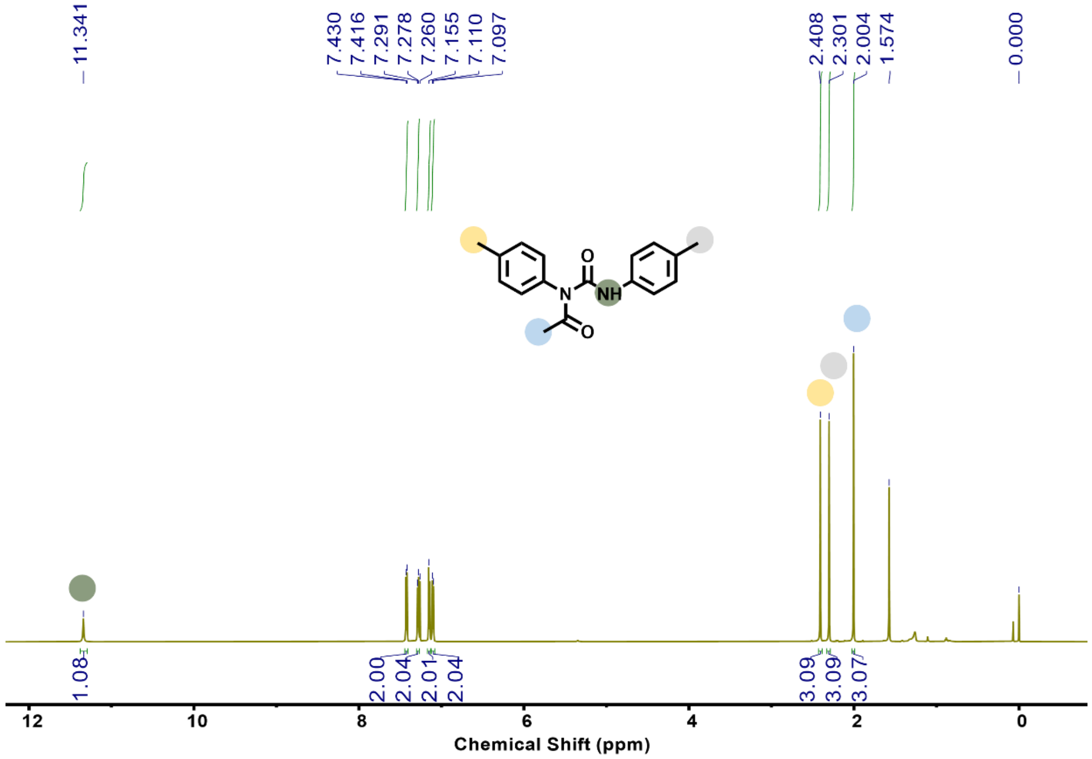

Supplementary Figure 10. ^{1}H NMR (600 MHz) of acetylate product of 1bb.

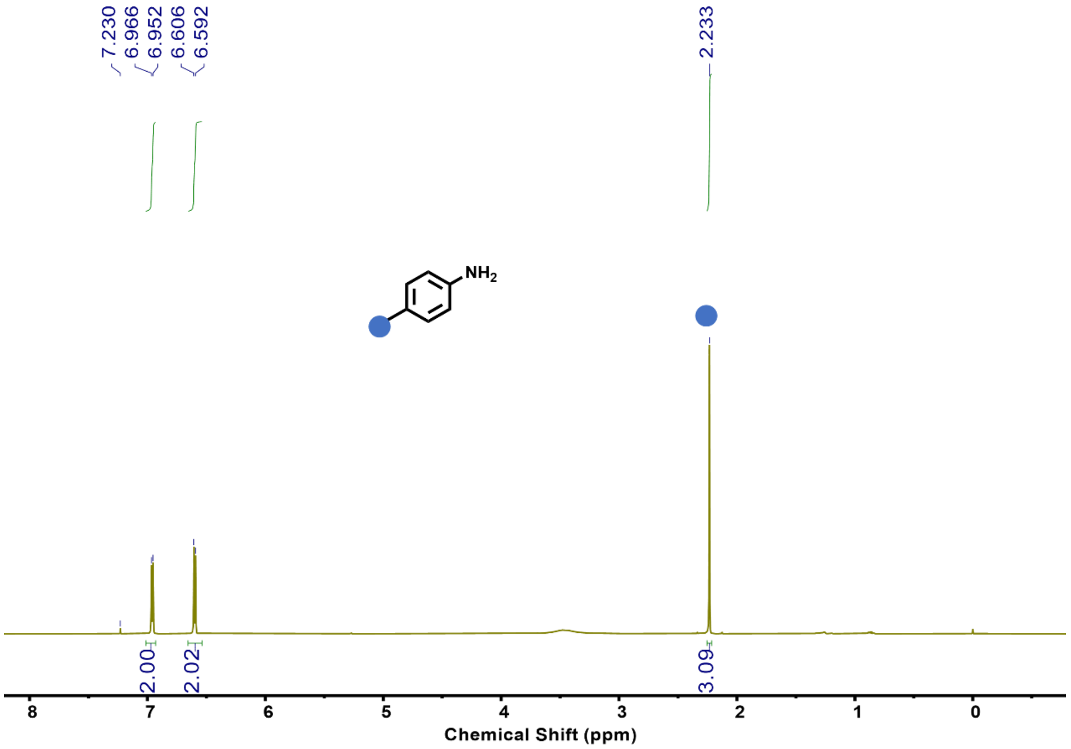

Supplementary Figure 11. ^{1}H NMR (600 MHz) of hydrolysis product of 1cc.

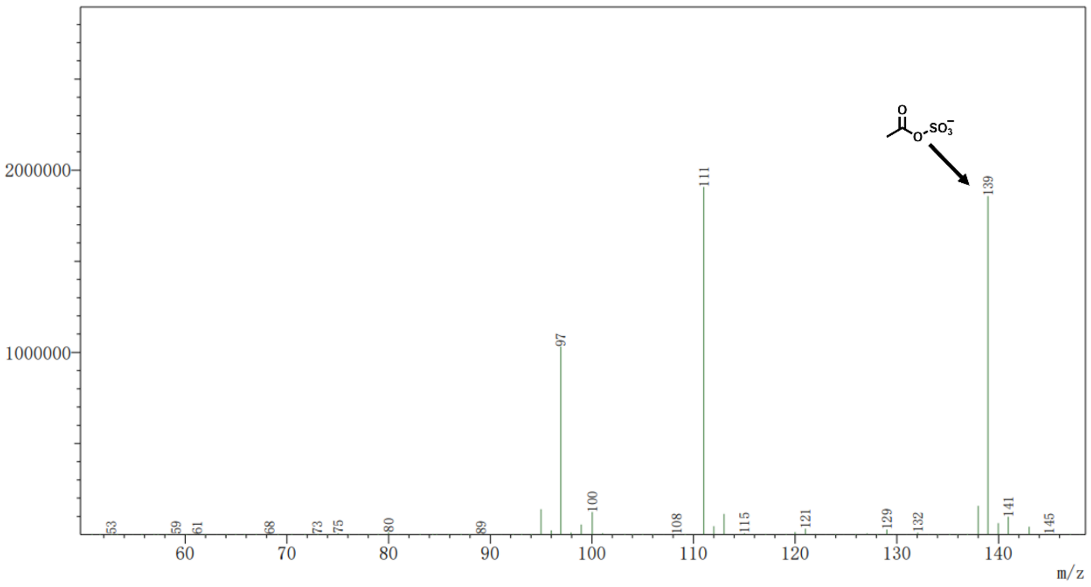

Supplementary Figure 12. LS-MS spectrum of acetic sulfuric anhydride ion.

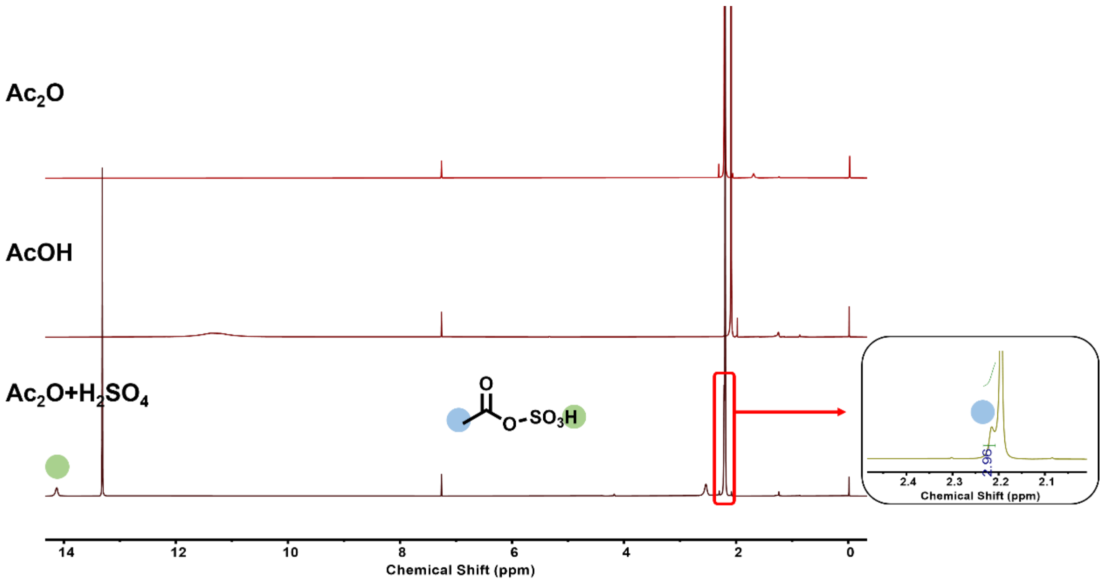

Supplementary Figure 13. ^{1}H NMR spectrum of the mixture of Ac_2O and H_2SO_4.

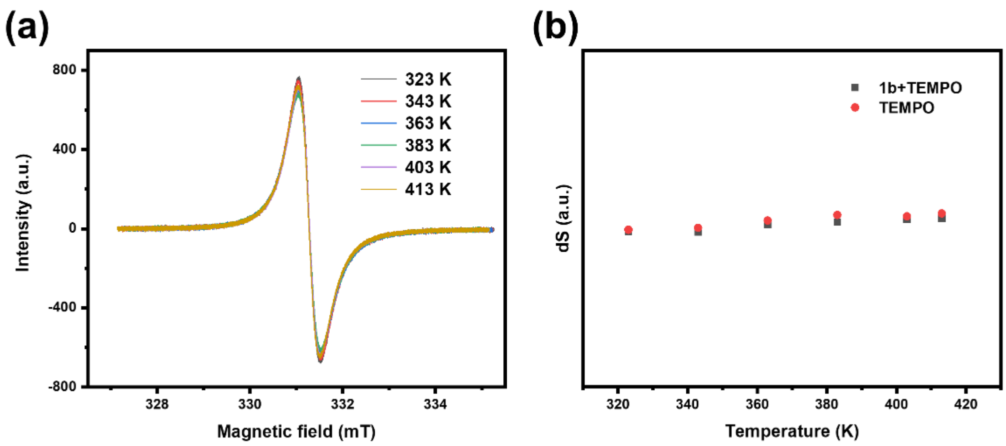

Supplementary Figure 14. (a) EPR spectrum of 1b and TEMPO and (b) comparison of integrated area with pure TEMPO at different temperatures.

4. Synthesis and Characterization of Polyureas

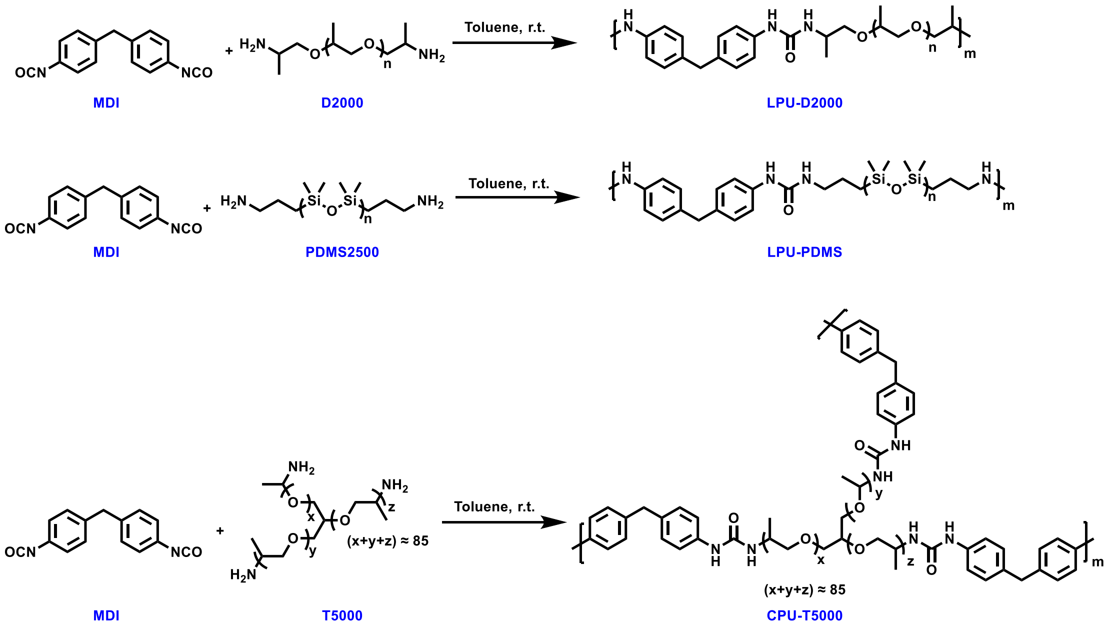

Supplementary Figure 15. Synthetic route of polyureas.

The synthesis of linear polyureas (LPU-D2000, LPU-PDMS) and cross-linked polyureas (CPU-T5000) followed similar procedures, with variations in the choice of polyamine macromonomer. Taking the synthesis of LPU-D2000 as a representative example: polyetheramine D2000 (2.0 g, 1.0 mmol) was dissolved in 20 mL of anhydrous toluene in a 100 mL three-necked round-bottom flask under a nitrogen atmosphere. MDI (0.25 g, 1.0 mmol), separately dissolved in 10 mL of anhydrous toluene, was added dropwise to the polyamine solution at 0 °C (approximately one drop per second) under constant stirring. Upon completion of the addition, the reaction mixture was stirred at room temperature for 2 h. For reactions involving PDMS-NH_2 and T5000, gelation occurred within seconds upon mixing with MDI. The resulting mixture was immediately cast onto a PTFE dish and dried in a vacuum oven at 80 °C for 6-12 h to yield LPU-D2000 films.

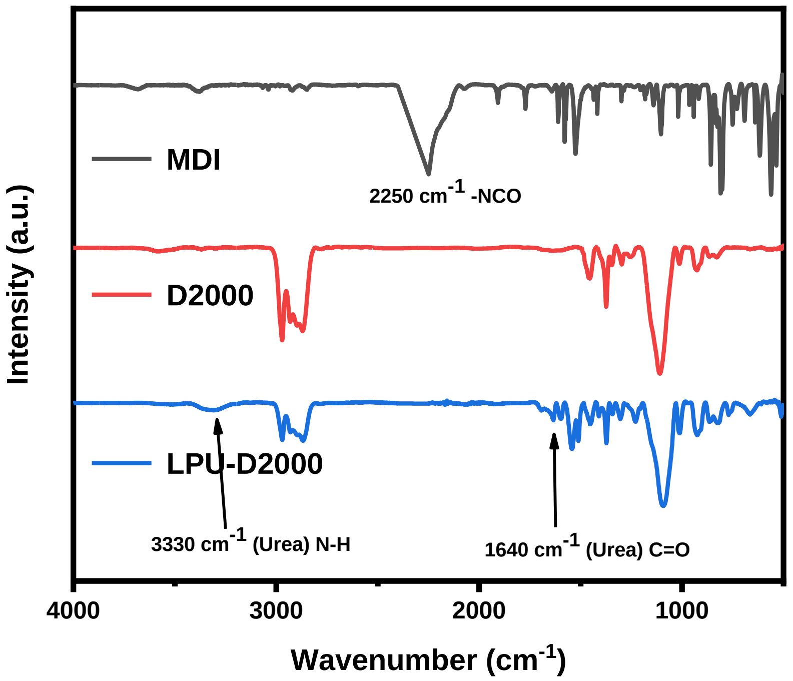

Supplementary Figure 16. FT-IR spectra of LPU-D2000 and monomers.

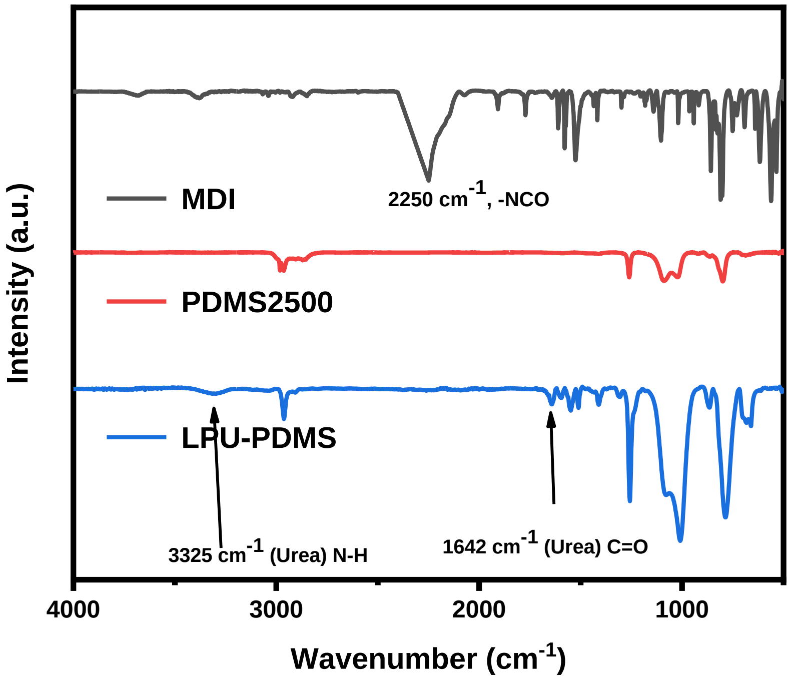

Supplementary Figure 17. FT-IR spectra of LPU-PDMS and monomers.

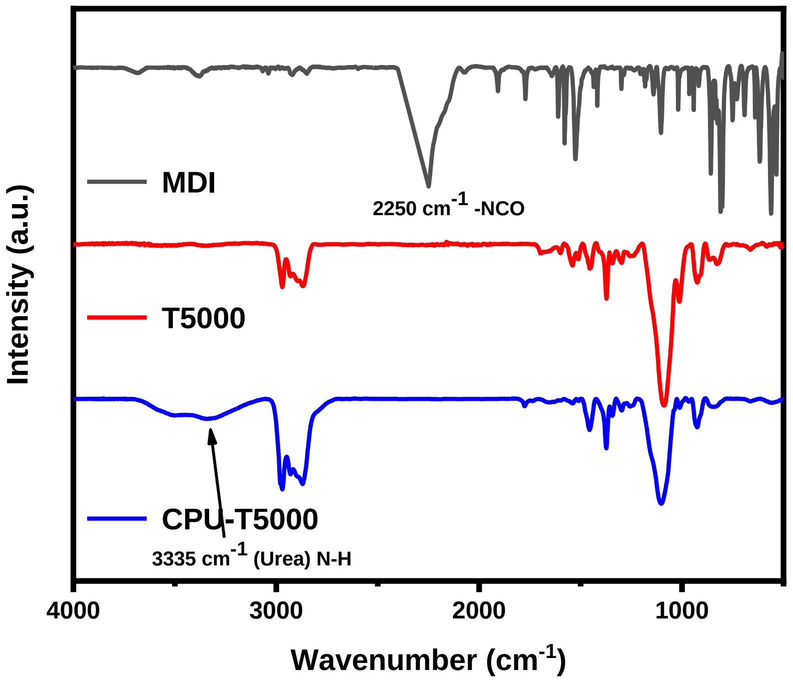

Supplementary Figure 18. FT-IR spectra of CPU-T5000 and monomers.

5. General Experimental Procedure of Depolymerization

5.1 General Depolymerization Methods

Used LPU-PDMS films (4.5 g) were cut into small pieces and immersed in 10.0 mL of *N*,*N*-dimethylacetamide (DMAc). Acetic anhydride (835.0 mg, 5 equiv.) and sulfuric acid (168.8 mg, 1 equiv.) were added, and the mixture was subjected to microwave heating at 100 °C for 20 min. After cooling to room temperature, DMAc and the byproduct acetic acid were recovered via vacuum distillation. The residue was treated with 20.0 mL of 1 M NaOH aqueous solution and 20.0 mL of ethanol, followed by refluxing at 80 °C for 2 h. Upon cooling, volatile components were removed under reduced pressure. The resulting mixture was diluted with dichloromethane (15.0 mL) and washed with saturated aqueous NaCl solution. The aqueous phase was extracted with dichloromethane (3 ×), and the combined organic layers were dried over anhydrous $MgSO_4$. After solvent removal under reduced pressure, the crude product was purified by column chromatography on alkaline silica gel (eluent: acetone/ethyl acetate = 1:5) to afford PDMS2500 as a transparent liquid with a yield of 60%. The depolymerization and separation procedures for LPU-D2000 and CPU-T5000 followed the same protocol as that of LPU-PDMS. Pure D2000 and T5000 were obtained with yields of 55% and 57%, respectively.

5.2 Characterization of Recycled Products

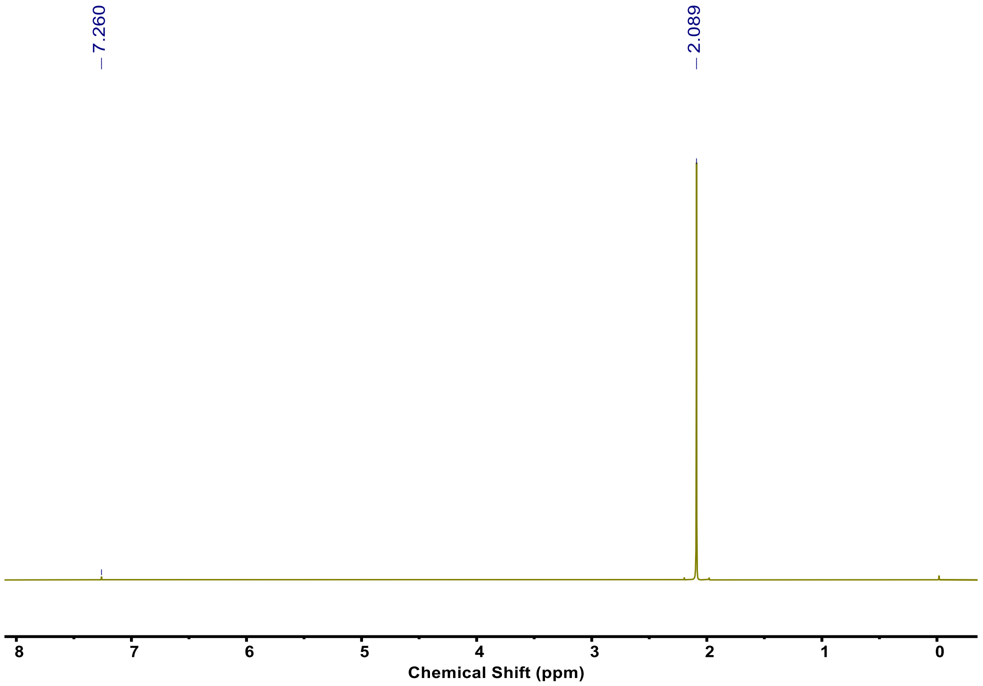

Supplementary Figure 19. ^{1}H NMR spectrum (600 MHz) of the recycled AcOH in $CDCl_3$.

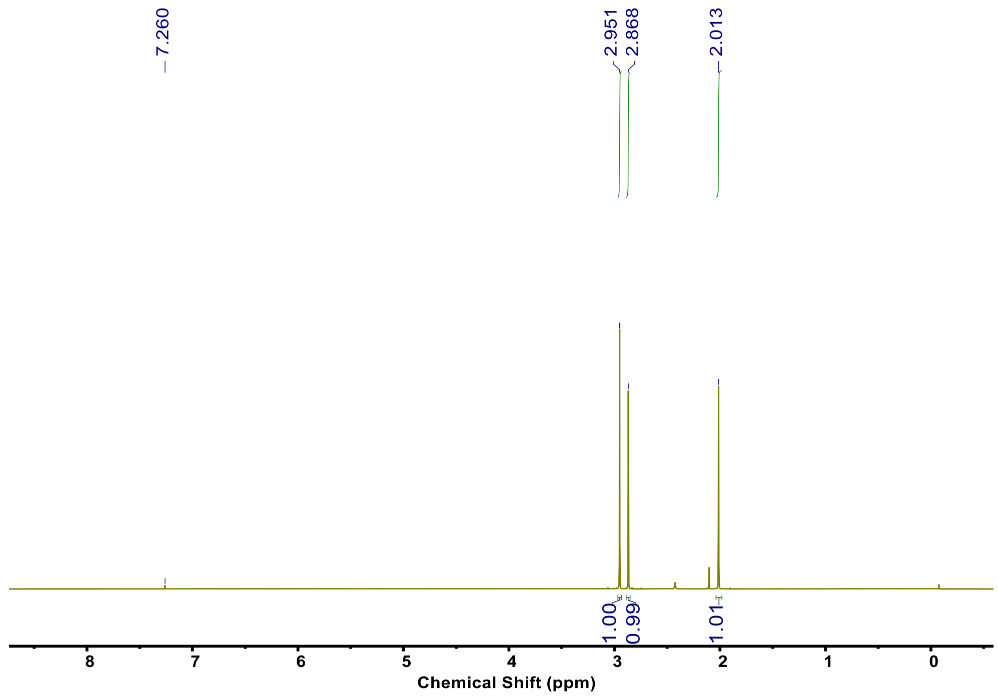

Supplementary Figure 20. ^{1}H NMR spectrum (600 MHz) of the recycled DMAc in $CDCl_3$.

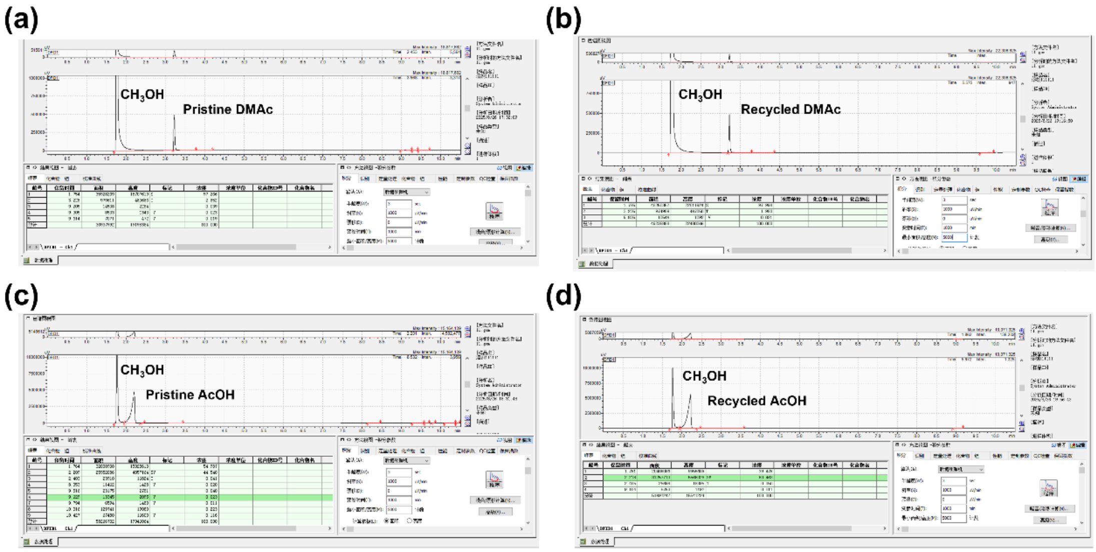

Supplementary Figure 21. GC spectra of the recycled DMAc and AcOH.

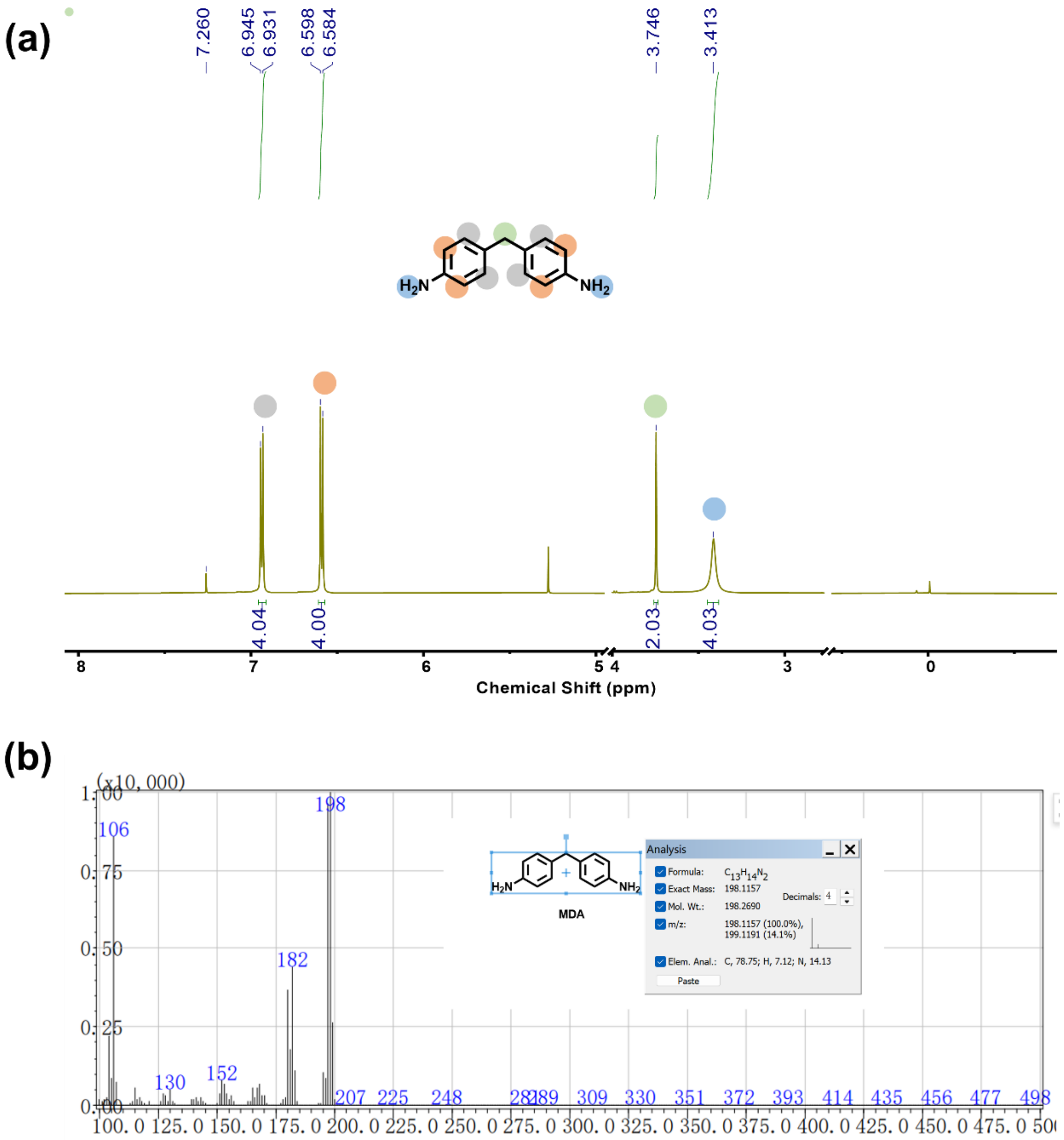

Supplementary Figure 22. (a) ^{1}H NMR and (b) GC-MS spectrum of MDA.

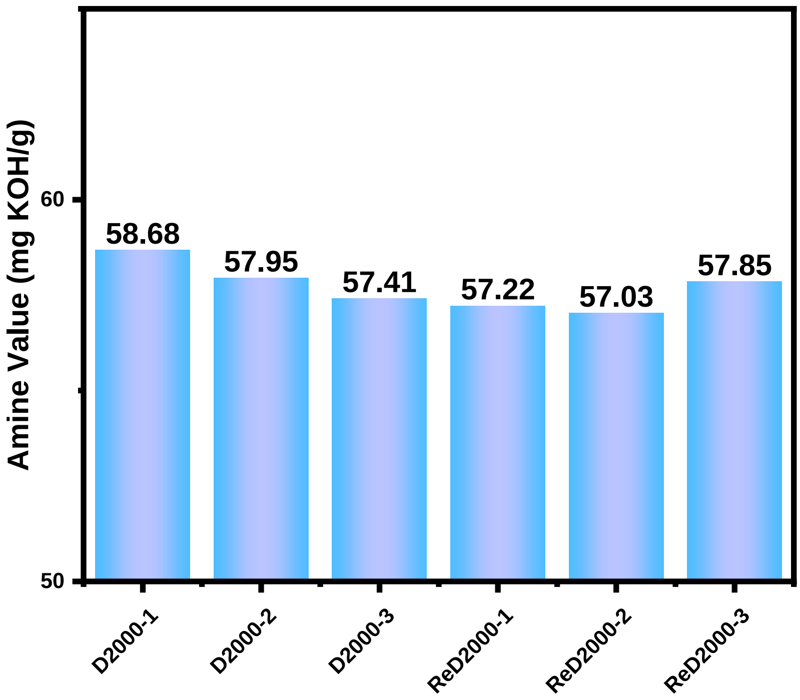

Supplementary Figure 23. Amine value titration of pristine and recycled D2000.

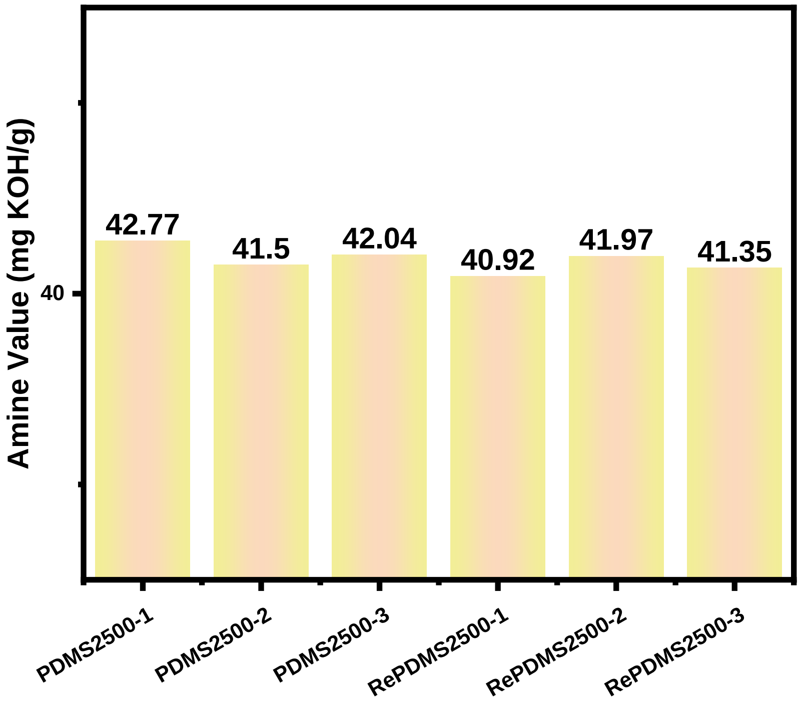

Supplementary Figure 24. Amine value titration of pristine and recycled PDMS2500.

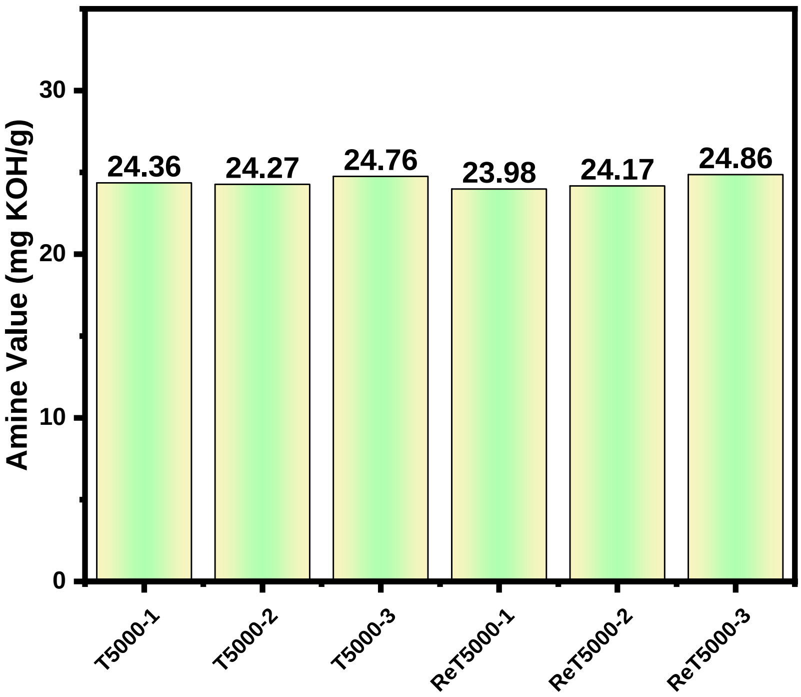

Supplementary Figure 25. Amine value titration of pristine and recycled T5000.

A bromophenol blue indicator solution (0.1 wt% in ethanol, 200 mL) was prepared. Polyetheramine D2000 (100.0 ± 0.1 mg) was accurately weighed into a 50 mL conical flask and dissolved in 15 mL of the indicator solution dissolve thoroughly, affording a homogeneous blue solution. This solution was titrated with standardized 1 M HCl. The endpoint was determined by a sharp chromatic transition from blue to yellow, with the HCl volume recorded to the nearest 2.0 μL. The amine value of polyamine monomer was estimated using theory by the following equation:

$$\text{Amine value} = \frac{v \times c \times 56.1}{m}$$

Where v is volume of hydrochloric acid consumed for titration (mL), c is hydrochloric acid concentration (mol/L), m is sample mass (g), 56.1 is molar mass of KOH (g/mol).

6. Mechanical Properties of Pristine and Recycled Polyureas

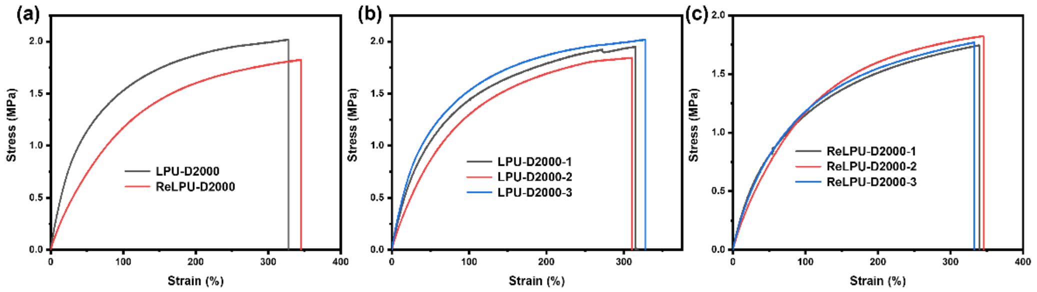

Supplementary Figure 26. Typical tensile curves of LPU-D2000.

Supplementary Table 3. Summarized mechanical properties of LPU-D2000.

	Breaking Strength (MPa)	Breaking Elongation (%)	Young's Modulus (KPa)
**LPU-D2000**	2.0 ±0.11	318 ±13	353 ±59
**ReLPU-D2000**	1.8 ±0.07	339 ±7	277 ±64

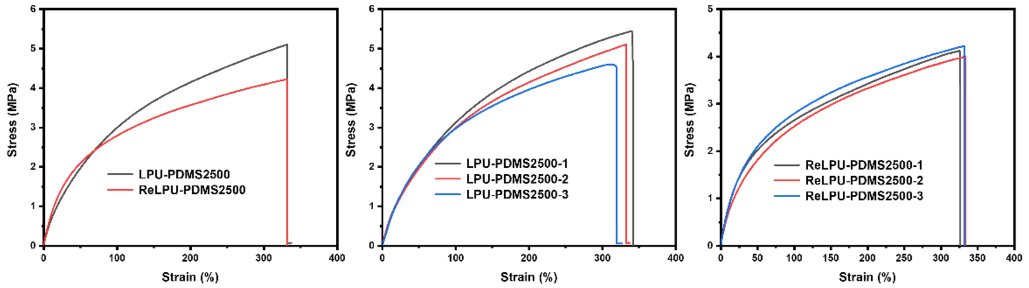

Supplementary Figure 27. Typical tensile curves of LPU-PDMS.

Supplementary Table 4. Summarized mechanical properties of LPU-PDMS.

	Breaking Strength (MPa)	Breaking Elongation (%)	Young's Modulus (KPa)
**LPU-PDMS**	5.1 ±0.55	331 ±18	729 ±10
**ReLPU-PDMS**	4.2 ±0.16	328 ±6	852 ±58

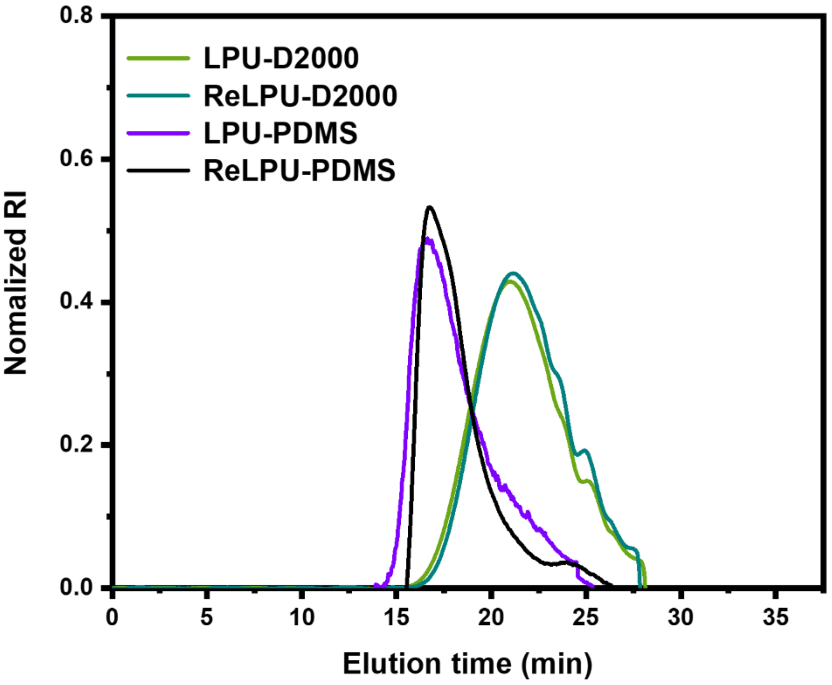

Sample	Mn (kDa)[a]	Đ (Mw/Mn)
LPU-D2000	32.6	1.78
ReLPU-D2000	30.2	1.80
LPU-PDMS	40.1	1.54
ReLPU-PDMS	38.9	1.64

[a] Calculated from GPC in *N*,*N*-Dimethylformamide.

Supplementary Figure 28. GPC traces of LPU-D2000, ReLPU-D2000, LPU-PDMS and ReLPU-PDMS.

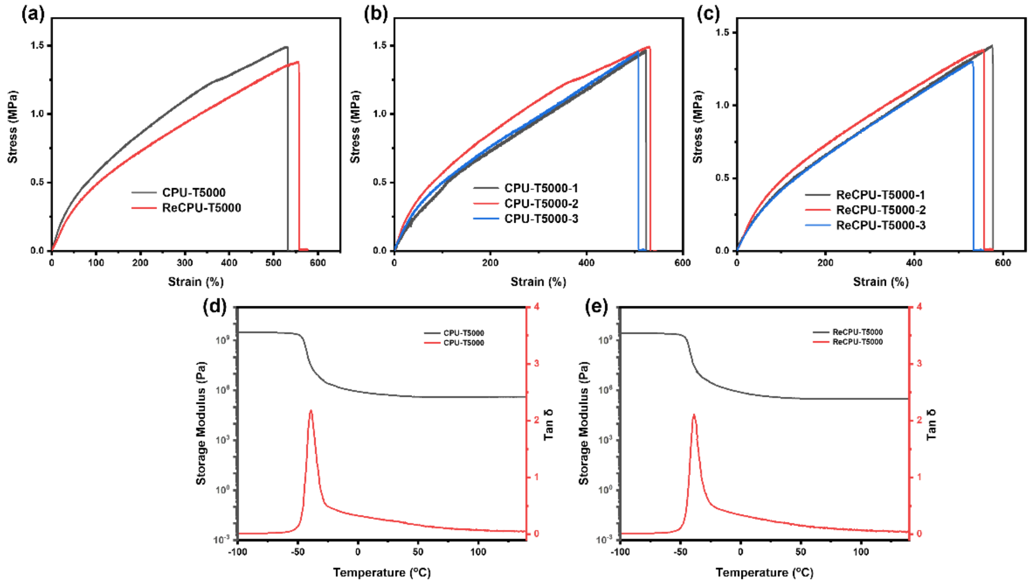

Supplementary Figure 29. (a)-(c) Typical tensile curves of CPU-T5000 and ReCPU-T5000, (d)-(f) DMA curves of CPU-T5000 and ReCPU-T5000.

Supplementary Table 5. Summarized mechanical properties of CPU-T5000.

	**Breaking Strength (MPa)**	**Breaking Elongation (%)**	**Young's Modulus (KPa)**	**Cross-linking Density^{a} (mol m^{-3})**
**CPU-T5000**	1.5 ±0.03	521 ±13	833 ±68	59.6
**ReCPU-T5000**	1.4 ±0.08	556 ±22	951 ±140	58.8

a: the cross-linking density (v) of polymer networks was estimated using rubber theory by the following equation:

$$\nu = \frac{E}{3RT}$$

where E is the plateau modulus of the rubbery network, R is the gas constant and T is the absolute temperature at which the modulus is measured.

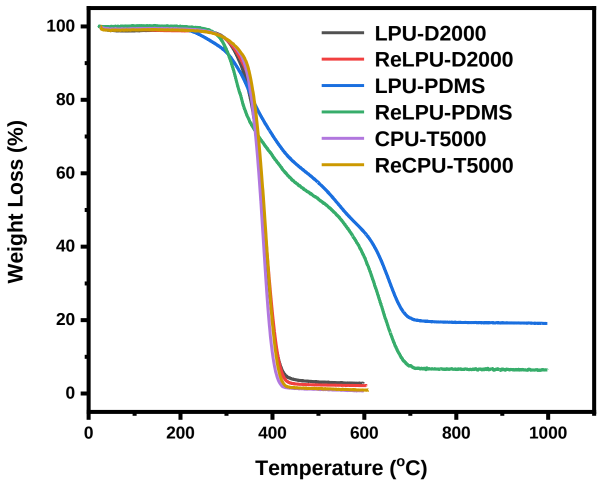

Supplementary Figure 30. TGA thermograms of pristine and recycled PU from room temperature to 600 ℃ or 1000 ℃. The experiment was performed at a heating rate of 20 ℃ min^{-1} under N_2 atmosphere.

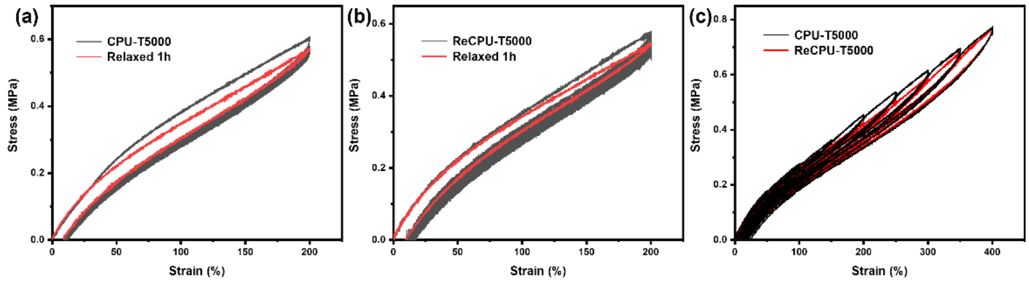

Supplementary Figure 31. (a-b) Representative loading-unloading curves of the CPU-5000 elastomer in cyclic tensile measurements with the fixed maximum strain of 200%. (c) Representative loading-unloading curves of the CPU-T5000 elastomer in cyclic tensile measurements with the stepwise strains from 0% to 400%.

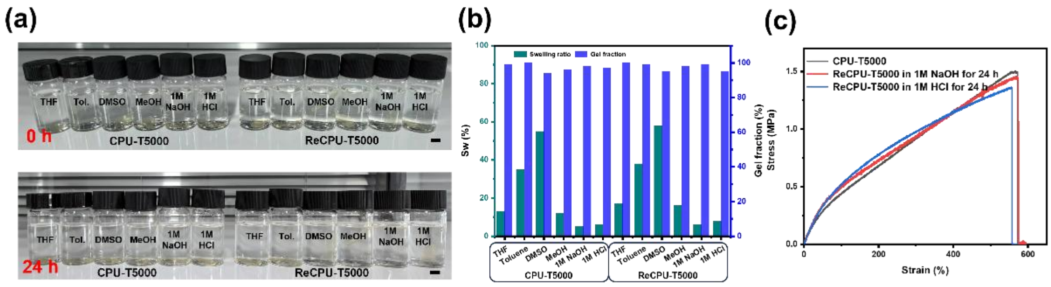

Supplementary Figure 32. (a) Digital photos of CPU-T5000 and ReCPU-T5000 before and after soaking in different solvents for 24 h. (Scale bar: 1 cm) (b) Mass swelling ratio and gel fraction of CPU-T5000 and ReCPU-T5000 in different solvents. (c) The stress–strain curves of CPU-T5000 and ReCPU-T5000 before and after soaking in 1M NaOH and 1M HCl aqueous solution for 24 h.

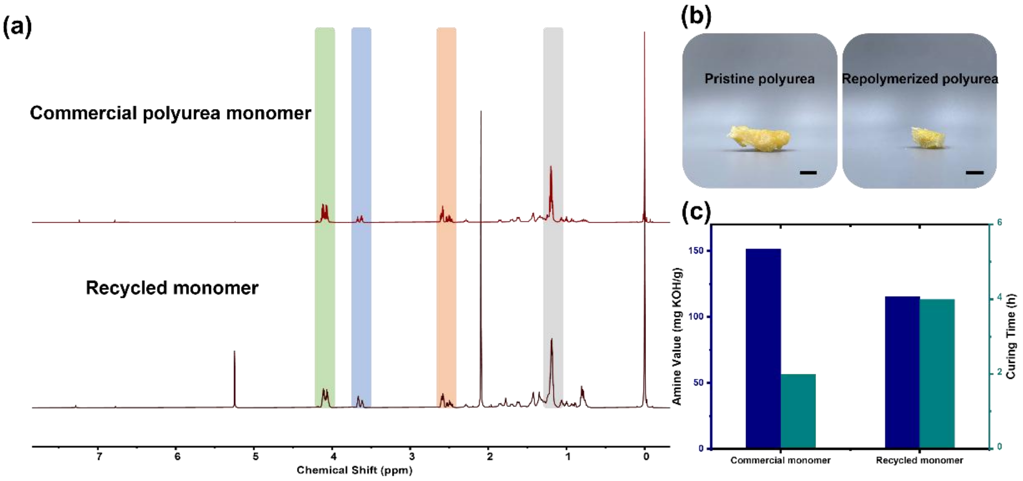

Supplementary Figure 33. (a) Digital photos of pristine commercial polyurea and repolymerized polyurea. (Scale bar: 1 cm) (b) Amine value and curing time of pristine commercial monomers and recycled monomers. (c) ^{1}H NMR spectra of pristine commercial monomers and recycled monomers.

7. Calculation data for Life Cycle and Techno-Economic Assessments

This comprehensive life cycle assessment (LCA) was conducted using openLCA software (v2.5). The study employed the Ecoinvent v3.11 database and the CML v4.8 impact assessment method to model the system. The functional unit was defined as the depolymerization of 4.5 g of polyurea. To ensure global relevance and comparability, background processes were modeled using a global average low-voltage electricity mix and market-based average transportation distances from the Ecoinvent database. A critical step involved scaling the experimental lab-scale energy consumption to an industrial context. This was achieved by researching comparable commercial-scale equipment and calculating the specific energy consumption per unit of processed material based on the industrial equipment's maximum processing capacity, power rating, and operational time, providing a more realistic estimate for a potential industrial-scale process.

Supplementary Table 6. Life cycle inventory of this work in total.

Name	Impact assessment result	Unit
Acidification	2.17E-02	kg SO2-Eq
Climate change	5.48E+00	kg CO2-Eq
Ecotoxicity: terrestrial	2.75E-02	kg 1,4-DCB-Eq
Energy resources: non-renewable	8.09E+01	MJ
Eutrophication	1.47E-01	kg PO4-Eq
Human toxicity	5.29E+00	kg 1,4-DCB-Eq
Ozone depletion	1.36E-05	kg CFC-11-Eq
Photochemical oxidant formation	2.73E-03	kg ethylene-Eq

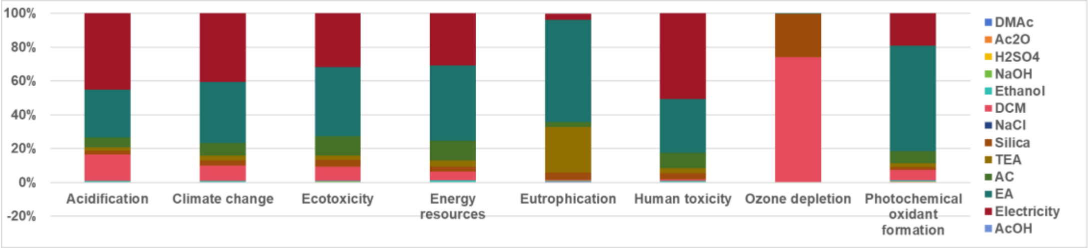

Supplementary Figure 34. Contribution of process components to the life cycle impacts of this work.

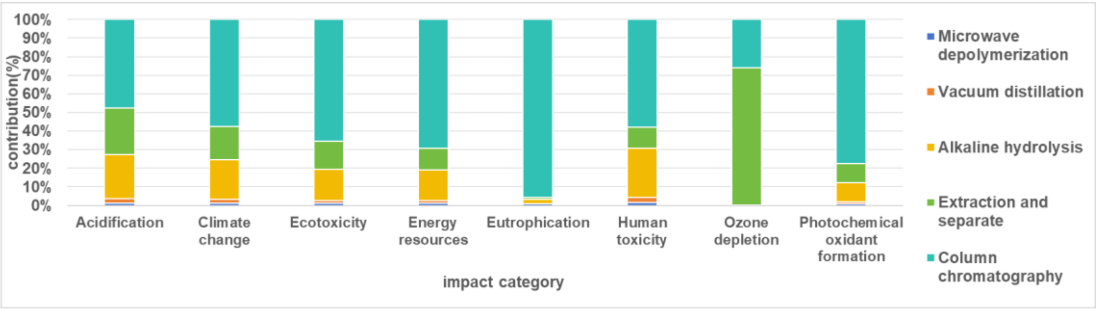

Supplementary Figure 35. Contribution of process components to the life cycle impacts of this work.

Supplementary Table 7. Contribution of process components to the life cycle impacts of this work.

Name	DMAc	Ac_2O	H_2SO_4	NaOH	Ethanol	DCM	AcOH
**Acidification**	1.41E-05	2.39E-05	3.21E-06	5.19E-06	9.87E-05	3.40E-03	-4.51E-06
**Climate change**	4.65E-03	8.59E-03	5.27E-05	1.12E-03	2.60E-02	5.10E-01	-1.78E-03
**Ecotoxicity**	2.64E-05	4.84E-05	5.08E-06	1.00E-05	1.48E-04	2.29E-03	-9.41E-06
**Energy resources**	8.06E-02	1.39E-01	6.45E-04	1.26E-02	6.04E-01	4.35E+00	-3.20E-02
**Eutrophication**	7.51E-04	5.25E-04	4.83E-06	2.80E-06	3.82E-04	3.12E-04	-2.23E-04
**Human toxicity**	4.37E-03	7.34E-03	1.30E-03	1.70E-03	2.36E-02	5.20E-02	-1.51E-03
**Ozone depletion**	7.04E-11	1.18E-10	5.64E-13	1.18E-10	4.37E-10	1.01E-05	-2.60E-11
**Photochemical oxidant formation**	2.08E-06	7.84E-06	1.34E-07	2.90E-07	2.03E-05	1.70E-04	-9.36E-07

Name	NaCl	Silica	TEA	AC	EA	Electricity
**Acidification**	7.82E-06	5.35E-04	3.72E-04	1.29E-03	6.14E-03	9.85E-03
**Climate change**	1.33E-03	1.56E-01	1.59E-01	4.13E-01	1.98E+00	2.23E+00
**Ecotoxicity**	3.36E-05	1.05E-03	7.24E-04	3.11E-03	1.13E-02	8.70E-03
**Energy resources**	1.43E-02	2.24E+00	2.98E+00	9.49E+00	3.60E+01	2.50E+01
**Eutrophication**	4.22E-06	6.51E-03	3.95E-02	4.64E-03	8.91E-02	5.49E-03
**Human toxicity**	4.52E-03	1.97E-01	1.45E-01	4.82E-01	1.68E+00	2.69E+00
**Ozone depletion**	1.10E-11	3.48E-06	1.97E-09	7.27E-09	3.13E-08	1.37E-08
**Photochemical oxidant formation**	4.65E-07	4.57E-05	6.02E-05	1.95E-04	1.71E-03	5.23E-04

Supplementary Table 8. Contribution of process components to the life cycle impacts of this work.

Name	Microwave depolymerization	Vacuum distillation	Alkaline hydrolysis	Extraction and separate	Column chromatography
**Acidification**	2.95E-04	5.13E-04	5.12E-03	5.44E-03	1.04E-02
**Climate change**	7.06E-02	1.15E-01	1.16E+00	9.70E-01	3.17E+00
**Ecotoxicity**	3.04E-04	4.48E-04	4.60E-03	4.12E-03	1.80E-02
**Energy resources**	8.62E-01	1.28E+00	1.33E+01	9.51E+00	5.59E+01
**Eutrophication**	1.42E-03	6.58E-05	3.19E-03	1.45E-03	1.41E-01
**Human toxicity**	8.22E-02	1.40E-01	1.40E+00	6.10E-01	3.06E+00
**Ozone depletion**	5.43E-10	6.96E-10	7.56E-09	1.01E-05	3.53E-06
**Photochemical oxidant formation**	2.35E-05	2.65E-05	2.87E-04	2.78E-04	2.11E-03

Supplementary Table 9. Contribution of process components to the life cycle impacts of this work compared with catalytic hydrogenolysis.

Name	This work	Catalytic hydrogenolysis	Unit
**Acidification**	2.17E-02	6.66E-02	kg SO2-Eq
**Climate change**	5.48E+00	1.49E+01	kg CO2-Eq
**Ecotoxicity**	2.75E-02	6.38E-02	kg 1,4-DCB-Eq
**Energy resources**	8.09E+01	1.79E+02	MJ
**Eutrophication**	1.47E-01	1.43E-01	kg PO4-Eq
**Human toxicity**	5.29E+00	1.53E+01	kg 1,4-DCB-Eq
**Ozone depletion**	1.36E-05	3.71E-05	kg CFC-11-Eq
**Photochemical oxidant formation**	2.73E-03	4.99E-03	kg ethylene-Eq

Supplementary Table 10. Contribution of process components to the life cycle impacts of catalytic hydrogenolysis [2].

Catalytic hydrogenolysis	Impact assessment result	Unit
**Acidification**	0.014810346	kg SO2-Eq
**Climate change**	3.305568846	kg CO2-Eq
**Ecotoxicity**	0.014181912	kg 1,4-DCB-Eq
**Energy resources**	39.68511476	MJ

Catalytic hydrogenolysis	Impact assessment result	Unit
**Eutrophication**	0.0317096	kg PO4-Eq
**Human toxicity**	3.405481912	kg 1,4-DCB-Eq
**Ozone depletion**	8.24119E-06	kg CFC-11-Eq
**Photochemical oxidant formation**	0.001108153	kg ethylene-Eq

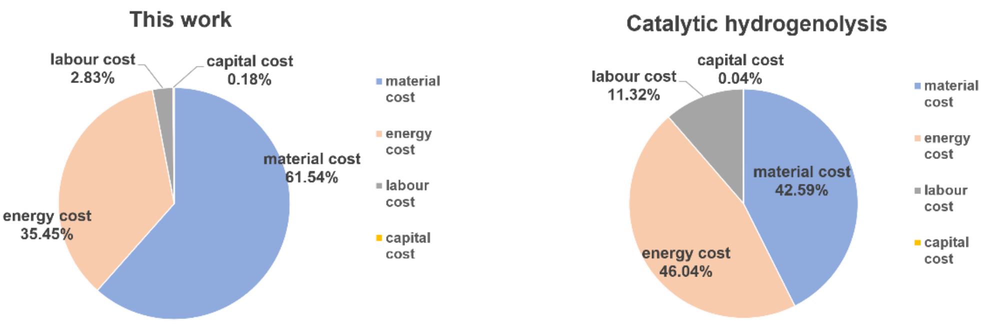

Supplementary Figure 36. Contribution of process components to the techno-economic analysis impacts of this work compared with catalytic hydrogenolysis.

Supplementary Table 11. Contribution of process components to the techno-economic analysis impacts of this work compared with catalytic hydrogenolysis.

	Material cost	Energy cost	Labour cost	Capital cost	Revenue
**This work**	7.0732	4.0744	0.3253	0.0211	67.0565
**Catalytic hydrogenolysis**	20.1750	21.8070	5.3632	0.0211	43.4241

8. Crystal Data of 1bb

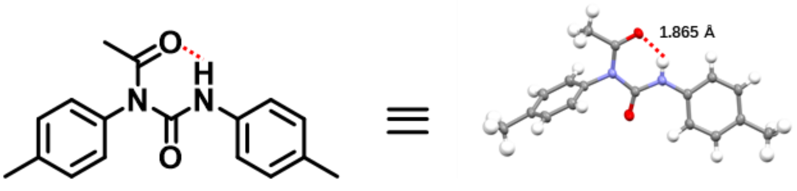

1bb

X-ray of 1bb
CCDC: 2456182

Supplementary Table 12 Crystal data and structure refinement for 1bb.

Identification code	1bb
Empirical formula	$C_{17}H_{18}N_2O_2$
Formula weight	282.33
Temperature/K	150.00
Crystal system	triclinic
Space group	P-1
a/Å	8.2762(3)
b/Å	9.6053(4)
c/Å	10.4504(4)
α/°	65.301(2)
β/°	83.864(2)
γ/°	76.582(2)
Volume/Å^3	734.11(5)
Z	2
ρ_{calc}g/cm^3	1.277
μ/mm^{-1}	0.679
F(000)	300.0
Crystal size/mm^3	0.32 × 0.29 × 0.25
Radiation	CuKα (λ = 1.54178)
2Θ range for data collection/°	9.316 to 136.436
Index ranges	$-9 \leq h \leq 9$, $-11 \leq k \leq 11$, $-11 \leq l \leq 12$
Reflections collected	11472
Independent reflections	2674 [R_{int} = 0.0806, R_{sigma} = 0.1017]
Data/restraints/parameters	2674/0/197
Goodness-of-fit on F^2	1.058
Final R indexes [I>=2σ (I)]	R_1 = 0.0381, wR_2 = 0.1035
Final R indexes [all data]	R_1 = 0.0820, wR_2 = 0.1088
Largest diff. peak/hole / e Å^{-3}	0.22/-0.18

Supplementary Table 13 Fractional Atomic Coordinates ($\times10^4$) and Equivalent Isotropic Displacement Parameters ($Å^2\times10^3$) for 1bb. U_{eq} is defined as 1/3 of the trace of the orthogonalised U_{IJ} tensor.

Atom	*x*	*y*	*z*	U(eq)
O2	10265.4(11)	1703.3(11)	9973.1(8)	29.5(2)
O1	8136.1(12)	2909.0(14)	6201.3(9)	41.3(3)
N2	10312.9(12)	1918.6(12)	7704.1(10)	24.4(2)
N1	7638.1(13)	2945.0(13)	8370.8(11)	26.7(2)
C9	11048.2(15)	1499.7(13)	8979.5(11)	23.9(3)
C11	11243.3(14)	1511.6(14)	6602.7(11)	24.3(3)
C6	5086.1(16)	4446.9(15)	6985.8(12)	29.1(3)
C8	8600.8(15)	2634.1(14)	7358.2(12)	26.2(3)
C5	5919.6(15)	3630.1(14)	8263.2(12)	24.8(3)
C4	5037.1(15)	3516.6(15)	9500.2(12)	27.4(3)
C12	11274.0(16)	75.3(15)	6594.1(12)	29.0(3)
C3	3359.6(16)	4197.3(15)	9465.1(12)	29.6(3)
C2	2509.5(15)	5021.8(14)	8194.5(13)	27.6(3)
C16	12002.7(16)	2605.5(15)	5529.0(12)	30.0(3)
C14	12838.6(15)	792.1(16)	4404.1(12)	28.6(3)
C7	3410.4(16)	5126.9(14)	6971.3(13)	29.0(3)
C15	12803.2(16)	2223.6(16)	4445.0(12)	31.9(3)
C1	685.6(16)	5768.4(16)	8129.2(14)	34.2(3)
C10	12877.8(15)	802.5(15)	9084.0(12)	28.8(3)
C13	12070.9(16)	-289.9(16)	5501.5(12)	31.2(3)
C17	13647.4(18)	415.7(19)	3190.7(14)	39.4(3)

Supplementary Table 14 Anisotropic Displacement Parameters ($Å^2\times10^3$) for 1bb. The Anisotropic displacement factor exponent takes the form: $-2\pi^2[h^2a^{*2}U_{11}+2hka^*b^*U_{12}+\ldots]$.

Atom	U_{11}	U_{22}	U_{33}	U_{23}	U_{13}	U_{12}
O2	28.5(5)	37.6(5)	23.0(4)	-15.8(3)	3.2(3)	-3.1(4)
O1	31.5(5)	62.5(7)	29.9(5)	-26.7(4)	-4.0(4)	7.2(5)
N2	23.1(5)	28.7(5)	22.1(5)	-12.6(4)	3.0(4)	-3.9(4)
N1	22.8(5)	33.7(6)	23.2(5)	-13.0(4)	2.2(4)	-3.2(4)
C9	26.1(6)	23.5(5)	22.5(5)	-10.1(4)	3.0(4)	-5.6(4)
C11	21.2(5)	31.0(6)	21.5(5)	-12.8(4)	2.0(4)	-3.9(4)

Atom	U_{11}	U_{22}	U_{33}	U_{23}	U_{13}	U_{12}
C6	27.4(6)	32.1(6)	25.2(6)	-11.7(5)	4.7(4)	-3.6(5)
C8	24.5(6)	29.2(6)	25.0(6)	-12.6(5)	2.0(4)	-3.4(5)
C5	23.0(6)	25.0(6)	27.4(6)	-12.3(4)	3.5(4)	-5.3(4)
C4	26.5(6)	29.8(6)	24.6(6)	-11.5(5)	1.1(4)	-3.1(5)
C12	30.9(6)	30.5(6)	26.1(6)	-12.7(5)	6.2(5)	-8.0(5)
C3	28.0(6)	32.8(6)	27.9(6)	-14.7(5)	7.6(5)	-5.1(5)
C2	24.8(6)	24.7(6)	33.6(6)	-13.6(5)	2.2(5)	-3.7(5)
C16	32.1(6)	31.3(6)	29.0(6)	-14.4(5)	5.4(5)	-9.7(5)
C14	22.5(6)	38.9(7)	24.5(6)	-15.4(5)	1.9(4)	-2.8(5)
C7	28.8(6)	28.1(6)	27.5(6)	-10.7(5)	-0.9(4)	-2.0(5)
C15	30.8(6)	38.1(7)	25.8(6)	-11.8(5)	7.6(5)	-11.3(5)
C1	26.6(7)	34.0(7)	39.3(7)	-15.6(5)	3.3(5)	-1.5(5)
C10	25.3(6)	33.4(6)	27.6(6)	-14.8(5)	0.2(4)	-1.4(5)
C13	33.5(7)	32.7(6)	29.7(6)	-17.0(5)	4.6(5)	-5.2(5)
C17	37.4(7)	53.4(9)	30.1(7)	-23.6(6)	8.1(5)	-5.6(6)

Supplementary Table 15 Bond Lengths for 1bb**.**

Atom	Atom	Length/Å	Atom	Atom	Length/Å
O2	C9	1.2261(14)	C6	C7	1.3878(18)
O1	C8	1.2102(15)	C5	C4	1.3906(17)
N2	C9	1.3857(16)	C4	C3	1.3878(17)
N2	C11	1.4552(14)	C12	C13	1.3886(17)
N2	C8	1.4355(16)	C3	C2	1.3955(18)
N1	C8	1.3481(16)	C2	C7	1.3869(17)
N1	C5	1.4165(16)	C2	C1	1.5087(17)
C9	C10	1.5023(16)	C16	C15	1.3895(18)

Atom	Atom	Length/Å	Atom	Atom	Length/Å
C11	C12	1.3777(18)	C14	C15	1.3869(19)
C11	C16	1.3887(17)	C14	C13	1.3977(18)
C6	C5	1.3942(18)	C14	C17	1.5087(16)

Supplementary Table 16 Bond Angles for 1bb.

Atom	Atom	Atom	Angle/°	Atom	Atom	Atom	Angle/°
C9	N2	C11	120.54(10)	C4	C5	N1	117.81(10)
C9	N2	C8	126.14(9)	C4	C5	C6	118.64(11)
C8	N2	C11	113.09(9)	C3	C4	C5	120.71(11)
C8	N1	C5	125.77(11)	C11	C12	C13	119.90(11)
O2	C9	N2	122.33(11)	C4	C3	C2	121.31(11)
O2	C9	C10	121.06(10)	C3	C2	C1	122.30(11)
N2	C9	C10	116.60(10)	C7	C2	C3	117.19(11)
C12	C11	N2	119.31(10)	C7	C2	C1	120.51(11)
C12	C11	C16	120.79(11)	C11	C16	C15	118.78(12)
C16	C11	N2	119.78(11)	C15	C14	C13	118.44(11)
C7	C6	C5	119.81(11)	C15	C14	C17	121.17(11)
O1	C8	N2	118.40(10)	C13	C14	C17	120.38(12)
O1	C8	N1	125.36(11)	C2	C7	C6	122.33(11)
N1	C8	N2	116.24(10)	C14	C15	C16	121.58(11)
C6	C5	N1	123.52(10)	C12	C13	C14	120.51(12)

Supplementary Table 17 Torsion Angles for 1bb.

A	B	C	D	Angle/°	A	B	C	D	Angle/°
N2	C11	C12	C13	176.04(11)	C8	N1	C5	C6	-17.6(2)
N2	C11	C16	C15	-176.33(11)	C8	N1	C5	C4	164.45(12)

A	B	C	D	Angle/°	A	B	C	D	Angle/°
N1	C5	C4	C3	178.33(11)	C5	N1	C8	O1	0.7(2)
C9	N2	C11	C12	87.09(14)	C5	N1	C8	N2	-179.35(10)
C9	N2	C11	C16	-96.79(14)	C5	C6	C7	C2	-0.3(2)
C9	N2	C8	O1	-178.83(12)	C5	C4	C3	C2	-0.4(2)
C9	N2	C8	N1	1.18(18)	C4	C3	C2	C7	0.20(19)
C11	N2	C9	O2	-173.65(10)	C4	C3	C2	C1	179.83(12)
C11	N2	C9	C10	7.44(16)	C12	C11	C16	C15	-0.27(19)
C11	N2	C8	O1	-4.35(16)	C3	C2	C7	C6	0.14(19)
C11	N2	C8	N1	175.66(10)	C16	C11	C12	C13	-0.04(19)
C11	C12	C13	C14	-0.3(2)	C7	C6	C5	N1	-177.87(11)
C11	C16	C15	C14	0.9(2)	C7	C6	C5	C4	0.11(19)
C6	C5	C4	C3	0.23(19)	C15	C14	C13	C12	0.86(19)
C8	N2	C9	O2	0.46(19)	C1	C2	C7	C6	-179.49(12)
C8	N2	C9	C10	-178.46(11)	C13	C14	C15	C16	-1.2(2)
C8	N2	C11	C12	-87.74(13)	C17	C14	C15	C16	177.34(12)
C8	N2	C11	C16	88.39(13)	C17	C14	C13	C12	-177.69(12)

Supplementary Table 18 Hydrogen Atom Coordinates (Å×10^4) and Isotropic Displacement Parameters (Å^2×10^3) for 1bb.

Atom	*x*	*y*	*z*	U(eq)
H6	5663.66	4537.84	6127.72	35
H4	5588.47	2967.11	10378.69	33
H12	10750.84	-664.08	7334.58	35
H3	2778.47	4100.18	10322.95	36
H16	11975.47	3596.75	5535.5	36
H7	2860.89	5684.03	6092.34	35
H15	13339.04	2960.78	3714.39	38

Atom	*x*	*y*	*z*	U(eq)
H1A	28.62	4953.2	8450.55	51
H1B	392.63	6496.33	7156.84	51
H1C	450.87	6340.72	8737.63	51
H10A	13484.06	1577.69	8412.18	43
H10B	13094.97	-123.02	8866.37	43
H10C	13247.79	497.89	10041.81	43
H13	12094.52	-1282.68	5499.88	37
H17A	12926.21	979.15	2367.14	59
H17B	13821.56	-714.06	3453.96	59
H17C	14719.63	737.46	2962.66	59
H1	8160(20)	2620(20)	9184(18)	40(4)

Crystal structure determination of [1bb].

Crystal Data for $C_{17}H_{18}N_2O_2$ (M =282.33 g/mol): triclinic, space group P-1 (no. 2), a = 8.2762(3) Å, b =9.6053(4) Å, c =10.4504(4) Å, α =65.301(2)°, β =83.864(2)°, γ =76.582(2)°, V =734.11(5) Å^3, Z =2, T =150.00 K, μ(CuKα) =0.679 mm^{-1}, $Dcalc$ =1.277 g/cm^3, 11472 reflections measured (9.316° ≤ 2Θ ≤ 136.436°), 2674 unique (R_{int} = 0.0806, R_{sigma} =0.1017) which were used in all calculations. The final R_1 was 0.0381 (I > 2σ(I)) and wR_2 was 0.1088 (all data).

9. Supplementary References

1. VOVK M V and SAMARAI L I. ChemInform Abstract: Synthesis of Aryl Isocyanates by Mild Thermolysis of N-Acyl-N, N′- diarylureas. 1992; **23**.
2. Ma Y, Jiang X, and Yin J, *et al.* Chemical upcycling of conventional polyureas into dynamic covalent poly(aminoketoenamide)s. *Angew Chem Int Ed*, 2023, 62; **3**: e202212870.
